# Maternal smoking during pregnancy increases the risk of gut microbiome-associated childhood overweight and obesity

**DOI:** 10.1080/19490976.2024.2323234

**Published:** 2024-03-04

**Authors:** Ye Peng, Hein M Tun, Siew C Ng, Hogan Kok-Fung Wai, Xi Zhang, Jaclyn Parks, Catherine J Field, Piush Mandhane, Theo J Moraes, Elinor Simons, Stuart E Turvey, Padmaja Subbarao, Jeffrey R Brook, Tim K Takaro, James A Scott, Francis KL Chan, Anita L Kozyrskyj

**Affiliations:** a The Jockey Club School of Public Health and Primary Care, Faculty of Medicine, The Chinese University of Hong Kong, Hong Kong, SAR, China; b Microbiota I-Center (MagIC), Hong Kong, SAR, China; c Li Ka Shing Institute of Health Sciences, Faculty of Medicine, The Chinese University of Hong Kong, Hong Kong, SAR, China; d Department of Medicine and Therapeutics, Institute of Digestive Disease, Faculty of Medicine, The Chinese University of Hong Kong, Hong Kong, SAR, China; e HKU-Pasteur Research Pole, School of Public Health, LKS Faculty of Medicine, The University of Hong Kong, Hong Kong, SAR, China; f Faculty of Health Sciences, Simon Fraser University, Burnaby, BC, Canada; g Cancer Control Research, BC Cancer Research Institute, Vancouver, BC, Canada; h Department of Agricultural, Food & Nutritional Science, University of Alberta, Edmonton, AB, Canada; i Department of Pediatrics, University of Alberta, Edmonton, AB, Canada; j Department of Pediatrics, Hospital for Sick Children, University of Toronto, Toronto, ON, Canada; k Department of Pediatrics and Child Health, Children’s Hospital Research Institute of Manitoba, University of Manitoba, Winnipeg, Canada; l Department of Pediatrics, Child and Family Research Institute, BC Children’s Hospital, University of British Columbia, Vancouver, BC, Canada; m Dalla Lana School of Public Health, University of Toronto, Toronto, ON, Canada

**Keywords:** Maternal smoking, gut microbiota, childhood obesity, butyrate production

## Abstract

Childhood obesity is linked to maternal smoking during pregnancy. Gut microbiota may partially mediate this association and could be potential targets for intervention; however, its role is understudied. We included 1,592 infants from the Canadian Healthy Infants Longitudinal Development Cohort. Data on environmental exposure and lifestyle factors were collected prenatally and throughout the first three years. Weight outcomes were measured at one and three years of age. Stool samples collected at 3 and 12 months were analyzed by sequencing the V4 region of 16S rRNA to profile microbial compositions and magnetic resonance spectroscopy to quantify the metabolites. We showed that quitting smoking during pregnancy did not lower the risk of offspring being overweight. However, exclusive breastfeeding until the third month of age may alleviate these risks. We also reported that maternal smoking during pregnancy significantly increased Firmicutes abundance and diversity. We further revealed that Firmicutes diversity mediates the elevated risk of childhood overweight and obesity linked to maternal prenatal smoking. This effect possibly occurs through excessive microbial butyrate production. These findings add to the evidence that women should quit smoking before their pregnancies to prevent microbiome-mediated childhood overweight and obesity risk, and indicate the potential obesogenic role of excessive butyrate production in early life.

## GRAPHICAL ABSTRACT

**Figure:**
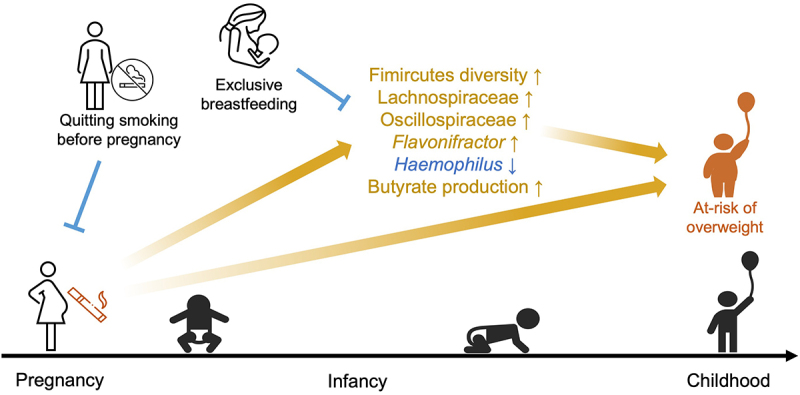

## Introduction

Childhood obesity is a growing concern, affecting over 18% of children and adolescents aged 5–19 globally by 2020. This figure rose rapidly, from just 4% in 1975.^1^ The increase in prevalence observed from 2010 to 2016 is also worth noting, especially in Southeast Asia, the Western Pacific, and Africa.^2,3^ Childhood obesity is linked to negative consequences, such as poorer health and self-esteem, being more likely to be bullied, and being more likely to become overweight or obese in adolescence and adulthood.^3^

Obesity is a multifactorial disease affected by a combination of factors, such as genetic predisposition, cesarean delivery, formula feeding, maternal overweight, dietary habits, reduced physical activity, medications, and secondhand smoke exposure.^4,5^ Among these factors, early life exposure to tobacco smoke and its relationship with the risk of overweight development is a critical concern, but this has not yet been well studied for obesity risk. A recent study reported an association between childhood exposure to parental smoking and an increased risk of life-course overweight or obesity (OWOB).^6^ An earlier meta-analysis also suggested that compared to paternal smoking, a direct intrauterine effect on childhood obesity is more prominent through maternal smoking during pregnancy.^7^ Furthermore, a dose-dependent association between childhood OWOB and maternal smoking during pregnancy has been reported.^8^

The gut microbiota, the complex and dynamic assembly of microorganisms residing in the gastrointestinal tract (GIT), has been associated with OWOB. Compared to their normal weight counterparts, the GIT of obese adults harbor a higher Firmicutes/Bacteroidetes (F/B) ratio and an enriched abundance of Firmicutes and Proteobacteria.^9,10^ Increased *Bacteroides fragilis* and *Staphylococcus* and depleted *Bifidobacterium* abundances have been repeatedly reported in association with childhood obesity across multiple cohorts.^11^ Experimental studies based on fecal microbiota transplantation or supplementation with microbiota-derived molecules have established a causal role of altered microbiota in the development of obesity.^9,12^ Previous studies have shown that the development of the gut microbiota in early life is influenced by various external and host-related factors.^13–18^ Being at the interface of external factors and host physiology and correlating with metabolic maturation,^13^ early-life gut microbiota was unsurprisingly recognized as a mediator in the paths toward childhood OWOB. Our previous study reported that Lachnospiraceae abundance in early infancy (~3 months old) mediated the household disinfectant-related increase in BMI z-scores at ages 1 year and 3 years in the Canadian Healthy Infant Longitudinal Development (CHILD) cohort.^14^ Similarly, enriched Lachnospiraceae abundance and microbial diversity in early infancy partially explain the increased risk of being overweight in formula-fed infants.^15^ Other studies within the CHILD cohort also revealed that Lachnospiraceae, Firmicutes richness, Ruminococcaceae, and *Bifidobacterium* were mediators of the pathway from birth mode to childhood overweight.^16,17^ Furthermore, a recent study in the same cohort reported that *Ruminococcus*, Enterobacteriaceae, and *Clostridium* in late infancy (12 months old) were enriched in children with a rapid BMI growth trajectory.^19^

The impact of maternal smoking on the infant gut microbiota has recently been discussed.^20^ Levin *et al* found that the abundance of *Akkermansia* and *Ruminococcus* at one month of age was positively associated with environmental tobacco smoke exposure in a US birth cohort.^21^ In another study, a German birth cohort study reported an association between maternal smoking during pregnancy and an increased abundance of *Blautia* and depleted *Faecalibacterium* abundance in 6-year-old children.^22^ However, no study has investigated the health consequences of maternal smoking-induced gut microbiota changes in children. Considering the role of infant gut microbiota in the development of childhood OWOB, the potential impact of maternal smoking during pregnancy on early infant gut microbiota and the weight status of children remains to be determined. Although a previous review proposed certain microbiota mediators^20^ in the path between maternal smoking during pregnancy and OWOB in offspring, a study with a large sample size with systematic consideration of potential confounders is necessary. We addressed these important questions in a large subset of the Canadian Healthy Infant Longitudinal Development (CHILD) Cohort study.

## Results

**Figure 1.**
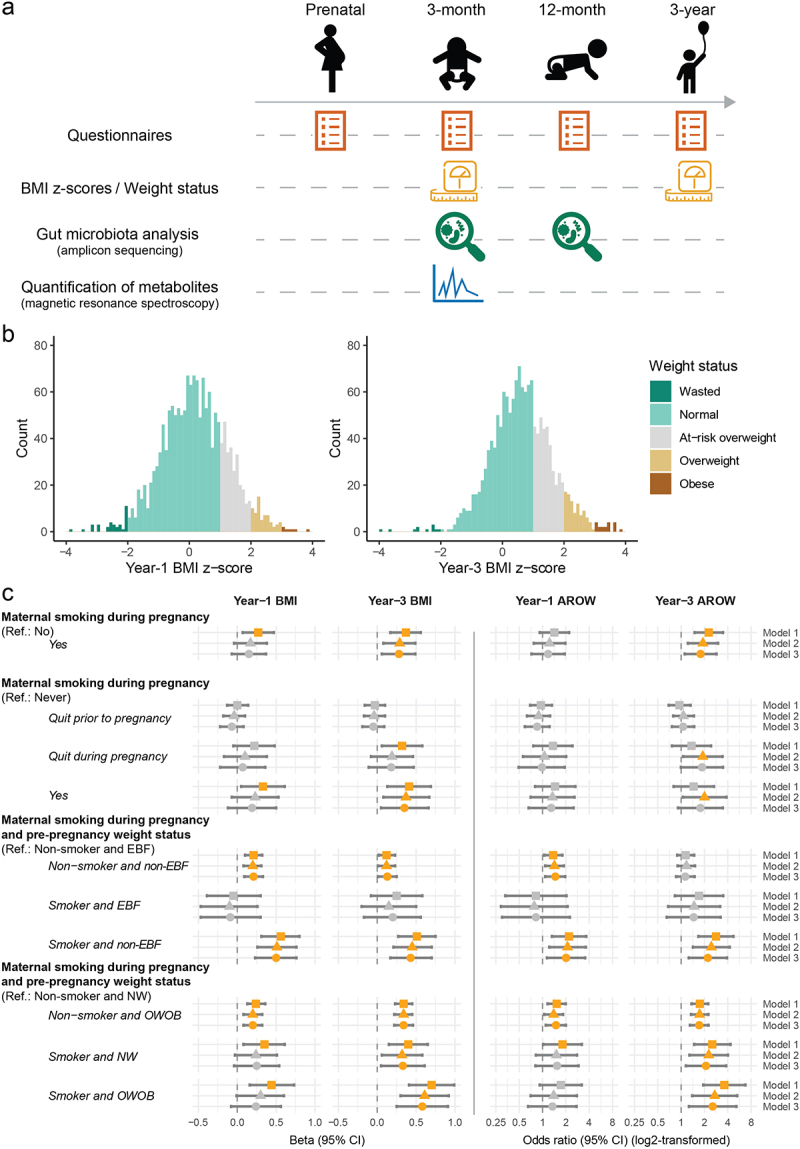
Schematic diagram for data collection and the association between maternal smoking during pregnancy and BMI z-scores of infants.

This study included a large subsample of 1,592 children with complete data on maternal smoking during pregnancy, BMI z-scores of children aged 1 year, and their gut microbiota data at ~3 months from the CHILD general population birth cohort (Figure 1a, Table 1). Of these children, 90.5% (*n* = 1,441) had BMI data at the age of 3 years, 87.2% (*n* = 1,389) had gut microbiota data at 12 months, and 79.6% (*n* = 1,268) had both.

(a) A schematic diagram for data collection. Questionnaires were collected prenatally and throughout the first three years of life. BMI scores were calculated based on weight and length/height data collected at age 1 year and age 3 years. Stool samples were collected at 3 and 12 months, and analyzed by amplicon sequencing of the V4 region of 16S rRNA and magnetic resonance spectroscopy for quantification of metabolites. (b) Distributions of BMI z-scores at 1 and 3 years. Weight status was categorized into “Wasted” (BMI z-score < -2), “Normal” (-2 ≤ BMI z-score ≤1), “At-risk overweight” (1 < BMI z-score ≤2), “Overweight” (2 < BMI z-score ≤3) and “Obese” (BMI z-score >3). (c) Association between maternal smoking during pregnancy and BMI z-scores of infants. Maternal race/ethnicity, socio-economic status, and maternal pre-pregnancy BMI (except in the models assessing joint effects of maternal BMI and smoking status) were adjusted in Model 1; prenatal and postnatal pet exposure, breastfeeding status at three months (except in the models assessing joint effects of maternal smoking status and breastfeeding status at three months), solid food introduction at three months, and household disinfectant use were additionally adjusted in Model 2; and prenatal diet calories, infant sex, birth mode and antibiotic use in the first three months were further adjusted in Model 3. Maternal education level was used as a proxy to the socio-economic status (SES) of the family. Maternal smoking status during pregnancy was categorized into 1) never smoker, 2) quit smoking before conception, 3) quit smoking during pregnancy, 4) cut the number of cigarettes during pregnancy, and 5) had the same number of cigarettes during pregnancy. In modeling of the effect of detailed maternal smoking statuses during pregnancy, cutting the number of cigarettes during pregnancy and having the same number of cigarettes during pregnancy were combined into one category (“Yes”) due to small sample sizes. These detailed statuses were also dichotomized into 1) “Yes/Smoker”: those who quit smoking during pregnancy, reduced the number of cigarettes, or had the same number of cigarettes (as before conception) during pregnancy; and 2) “No/Non-smoker”: those who quit smoking before conception, or never smoked. NW, normal weight. OWOB, overweight or obese. AROW, at-risk of overweight.

**Table 1.** Characteristics of the study subjects by exposure to maternal smoking during pregnancy.

	Overall (*N* = 1592)	Non-smoke exposed (*N* = 1458)	Smoke exposed (*N* = 134)	*P* value
***Continuous variables***				
Cotinine concentration (ng/mL, log2-transformed)	−3.54 (−4.51, −1.94)	−3.63 (−4.56, −2.27)	1.03 (−1.59, 2.98)	<0.001
Hydroxycotinine concentration (ng/mL, log2-transformed)	−2.63 (−3.8, −0.96)	−2.82 (−3.88, −1.31)	2.61 (−0.07, 4.43)	<0.001
Maternal education level	7 (5, 7)	7 (5, 7)	5 (3, 6)	<0.001
Maternal pre-pregnancy BMI [mean (sd)]	25.11 (5.63)	24.96 (5.47)	26.67 (6.99)	0.008
Prenatal calories intake	1957.5 (1566.4, 2403.9)	1954.0 (1570.6, 2394.2)	2029.2 (1517.3, 2522.8)	0.516
***Categorical variables***				
Breastfeeding status at 3 months				<0.001
Exclusive	904 (56.9)	864 (59.3)	40 (30.1)	
Partial	429 (27)	394 (27.1)	35 (26.3)	
Zero	256 (16.1)	198 (13.6)	58 (43.6)	
Pet exposure in the first 18 weeks				<0.001
No	709 (46.4)	680 (48.3)	29 (24)	
Only prenatal	118 (7.7)	100 (7.1)	18 (14.9)	
Only Postnatal	16 (1)	14 (1)	2 (1.7)	
Both Pre and Postnatal	686 (44.9)	614 (43.6)	72 (59.5)	
Solid food introduction at 3 months				0.002
No	1537 (97.3)	1414 (97.7)	123 (92.5)	
Yes	43 (2.7)	33 (2.3)	10 (7.5)	
Birth mode				0.86
Vaginal IAP-	834 (53.9)	763 (53.9)	71 (54.2)	
Vaginal IAP+	346 (22.4)	319 (22.5)	27 (20.6)	
C-Section (Elective)	149 (9.6)	134 (9.5)	15 (11.5)	
C-Section (Emergency)	218 (14.1)	200 (14.1)	18 (13.7)	
Direct antibiotic use in the first 3 months				0.136
No	1353 (91.4)	1241 (91.7)	112 (87.5)	
Yes	128 (8.6)	112 (8.3)	16 (12.5)	
Any postnatal disinfectant use				<0.001
No	723 (46.7)	685 (48.1)	40 (31.7)	
Yes	825 (53.3)	739 (51.9)	86 (68.3)	
Sex				0.366
Female	741 (46.5)	684 (46.9)	57 (42.5)	
Male	851 (53.5)	774 (53.1)	77 (57.5)	
Maternal race				<0.001
Asian	236 (14.8)	230 (15.8)	6 (4.5)	
Caucasian	1205 (75.8)	1103 (75.7)	102 (76.7)	
Other	149 (9.4)	124 (8.5)	25 (18.8)	
Number of adults at home				<0.001
1	33 (2.1)	23 (1.6)	10 (7.8)	
2	1274 (81.2)	1176 (81.6)	98 (76.6)	
≥3	262 (16.7)	242 (16.8)	20 (15.6)	
Having siblings				0.581
Yes	783 (50)	716 (49.8)	67 (52.3)	
Weight status in year 1				0.031
Normal	1209 (75.9)	1116 (76.5)	93 (69.4)	
At-risk overweight	277 (17.4)	245 (16.8)	32 (23.9)	
Overweight	65 (4.1)	56 (3.8)	9 (6.7)	
Obese	7 (0.4)	7 (0.5)	0 (0)	
Wasted	34 (2.1)	34 (2.3)	0 (0)	
Weight status in year 3				<0.001
Normal	963 (66.8)	907 (68.2)	56 (50)	
At-risk overweight	354 (24.6)	314 (23.6)	40 (35.7)	
Overweight	93 (6.5)	79 (5.9)	14 (12.5)	
Obese	19 (1.3)	17 (1.3)	2 (1.8)	
Wasted	12 (0.8)	12 (0.9)	0 (0)	
At-risk overweight or above in year 1				0.022
Yes	349 (22.4)	308 (21.6)	41 (30.6)	
At-risk overweight or above in year 3				<0.001
Yes	466 (32.6)	410 (31.1)	56 (50)	

*p* values for comparisons of continuous data between groups were given by Wilcoxon’s tests or *t* tests where appropriate; *p* values for comparisons of categorical data between groups were given by Fisher’s exact tests.

In years 1 and 3, most children had a normal weight (*n* = 1,209; 75.9% and *n* = 963; 66.8%), followed by those who were at risk of being overweight (AROW, *n* = 277; 17.4% and *n* = 354; 24.6%), and those who were overweight or obese (OWOB, *n* = 72; 4.5% and *n* = 112; 7.8%). Only a small proportion of children were wasted, with a BMI z-score of < −2 (year 1, *n* = 34; 2.1%; year 3, *n* = 12; 0.8%). Furthermore, the overall BMI z-score distribution shifted toward higher values at year 3 compared to year 1 (mean ± s.d.:0.62 ± 0.99 vs. 0.18 ± 1.06, *p* < .001) (Figure 1b).

### Maternal smoking during pregnancy increases the risk of being overweight at 1 and 3 years

Over 91% (*n* = 1,458) of the mothers either never smoked (*n* = 1,151; 72.3%) or quit smoking prior to pregnancy (*n* = 307; 19.3%) [median (IQR) years prior to conception:5.6 (2.7, 9.5)]. For the remaining 8.4%, half of them quit smoking (*n* = 68; 4.3%) and the other half cut (*n* = 54; 3.4%) or had the same number of cigarettes during pregnancy (*n* = 12; 0.8%). These self-reported data on smoking prior to and/or during pregnancy were highly correlated with concentrations of infant urinary nicotine metabolites, such as cotinine and *trans*-3’-hydroxycotinine, measured at the age of 3 months (Methods, Figure S1a-b), regardless of postnatal secondhand smoke exposure (Figure S2). Of note, the earlier mothers quit smoking prior to and during pregnancy, the lower the concentrations of these nicotine metabolites in the children’s urine (years quitting smoking prior to pregnancy: cotinine, Spearman’s Rho = −0.17, *p* = .006; hydroxycotinine, Spearman’s Rho = −0.28, *p* < 0.001) (weeks of pregnancy when quitting smoking: cotinine, Spearman’s Rho = 0.29, *p* = .048; hydroxycotinine, Spearman’s Rho = 0.22, *p* = .130) (Figure S1c-d). Food intake in 3-year-olds did not differ between children with and without exposure to maternal tobacco smoking during pregnancy, nor between children with normal weight and children who were AROW or OWOB in years 1 and 3 (Table S1).

To quantify the associations between maternal smoking status and childhood weight outcomes, we used generalized linear models with different adjustment levels (Methods, Figure S3, Table 1, Table S2). Compared to their non-exposed counterparts, children with exposure to maternal smoking during pregnancy had higher BMI z-scores at 1 year of age [Model 1: β = 0.27, 95% confidence interval (CI) 0.07–0.47], although it was not statistically significant with further adjustments (Figure 1c). This association was more evident for BMI z-scores at 3 years, even in the most adjusted model (Model 3: β = 0.28, 95% CI 0.06–0.49) (Figure 1c). They also had significantly higher odds of becoming AROW or OWOB at 3 years of age (Model 3: OR 1.78, 95% CI 1.11–2.86) but not at 1 year of age (Figure 1c). Interestingly, further analyses revealed that maternal smoking during pregnancy was positively associated with BMI z-score. Specifically, when compared with the children of nonsmoking mothers, quitting smoking prior to pregnancy was not associated with BMI z-scores of offspring at both ages 1 and 3 years (Figure 1c). On the other hand, quitting smoking during pregnancy was associated with increased BMI z-scores at age 3 years of age in the crude model (Model 1: β = 0.32, 95% CI 0.06–0.58); the association became not significant in Models 2 and 3, probably attributed to breastfeeding status being adjusted as a covariate, which may potentially serve as a mediator in the causal pathway. In line with this trend, remaining smoking during pregnancy with reduced or the same number of cigarettes, was associated with the highest BMI z-scores at 3 years of age (Model 3: β = 0.35, 95% CI 0.05–0.66). In addition, infants had a higher odd of becoming at-risk of overweight if they were born to a mother who quit smoking during pregnancy (Model 2: OR = 1.90, 95% CI 1.06–3.42) or smoked cigarettes during pregnancy (Model 2: OR = 2.00, 95% CI 1.05–3.84) (Figure 1c).

We next examined the joint effect of maternal smoking status during pregnancy and breastfeeding status at three months on childhood weight outcomes, given its influence on the above associations. Compared to children of nonsmoking mothers and exclusively breastfed (EBF) at three months, those of nonsmoker mothers but not exclusively breastfed (non-EBF) had higher BMI z-scores at both age 1 year (Model 3: β = 0.21, 95% CI 0.09–0.33) and age 3 years (Model 3: β = 0.13, 95% CI 0.01–0.25), as well as higher odds of being at risk of being overweight at age 1 year (Model 3: OR = 1.46, 95% CI 1.08–1.96) (Figure 1c). Alarmingly, within the non-EBF stratum, those with exposure to maternal smoking during pregnancy had even higher BMI z-scores and odds of becoming at risk of being overweight at both year 1 (BMI z-score Model 3: β = 0.50, 95% CI 0.23–0.76; AROW or OWOB Model 3: OR = 2.01, 95% CI 1.14–3.55) and year 3 (BMI z-score Model 3: β = 0.43, 95% CI 0.17–0.70; AROW or OWOB Model 3: OR = 2.21, 95% CI 1.25–3.90) (Figure 1c). In contrast, those with maternal smoking exposure but exclusively breastfed at three months did not show significantly increased risks as was observed in the non-EBF stratum (*p* > .05) (Figure 1c). However, given the broad confidence intervals observed in this group, a larger sample size may be required to robustly demonstrate breastfeeding’s effect in attenuating the smoke exposure-associated risks. As revealed by an interaction analysis, maternal smoking during pregnancy was significantly associated for all weight outcomes (except year-1 AROW) only within the stratum of non-EBF infants (Table S3). In addition, there were no multiplicative or additive interactions between these two factors (Table S3).

Since maternal pre-pregnancy weight status is a well-known risk factor for childhood overweight, we also tested the joint effect of maternal pre-pregnancy weight and maternal smoking status during pregnancy. When considering children of normal-weight mothers who did not smoke during pregnancy as the reference group, children of normal-weight mothers who smoked during pregnancy had a higher risk of becoming overweight as opposed to children of nonsmoking OWOB mothers at the age of 3 years (Model 3: OR 2.09, 95% CI 1.15–3.80) (Figure 1c). Additionally, being the highest-risk group, children of smoking OWOB mother had 2.55 (1.26, 5.16) higher odds of being at risk of being overweight at 3 years of age (Model 3) (Figure 1c). In an interaction analysis, maternal smoking during pregnancy was significantly associated weight outcomes at 3 years within the stratum of normal weight mothers; meanwhile, no multiplicative or additive interactions were observed between these two factors (Table S4).

For early life tobacco exposure, it is important to dissect its effect into the direct intrauterine effect due to maternal smoking during pregnancy and the effect of postnatal secondhand smoke exposure. Compared to postnatal secondhand smoke exposure, maternal smoking during pregnancy had a greater impact on the BMI z-scores of the offspring (Figure S4, Table S5). Furthermore, the urine levels of nicotine metabolites in offspring at the age of 3 months were positively correlated with BMI z-scores (Pearson’s correlation tests, *p* < 0.05), but not with AROW or OWOB in both years 1 and 3 (*p* > .05, Table S6).

### Maternal smoking during pregnancy is associated with altered gut microbiota in offspring

**Figure 2.**
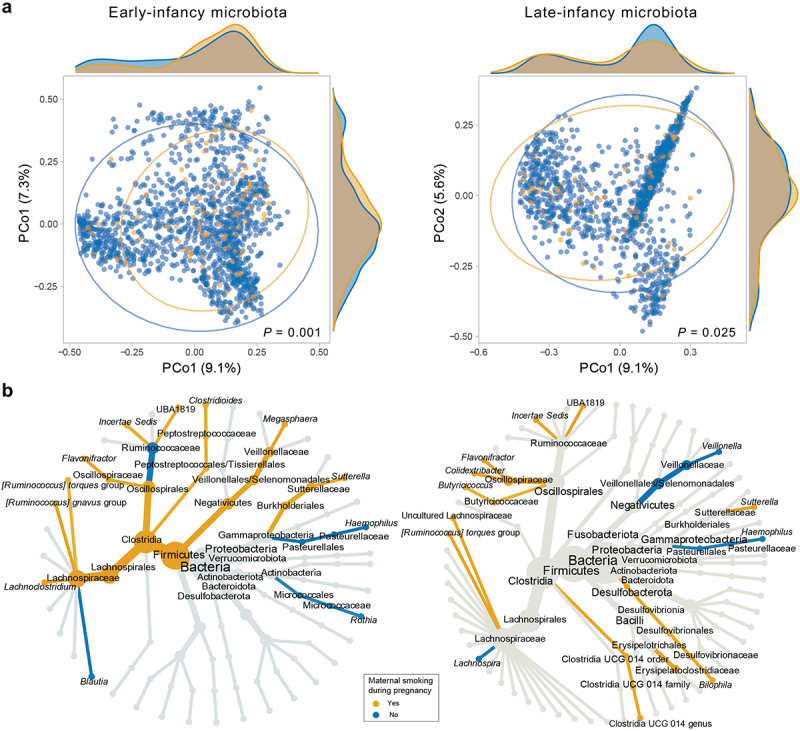
Associations between maternal smoking during pregnancy and profiles of gut microbiota in infancy.

**Figure 3.**
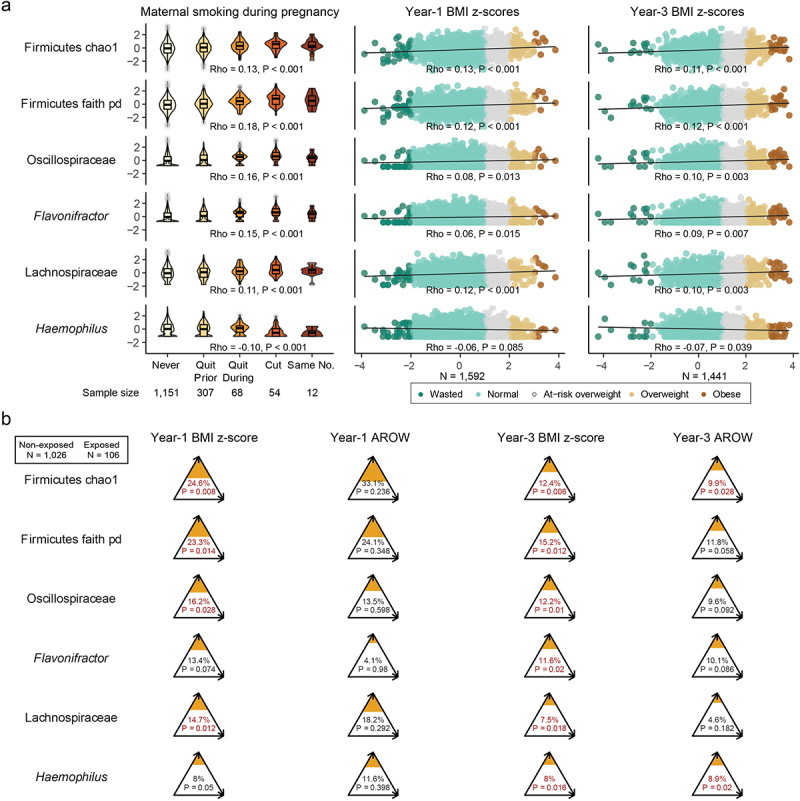
Early-infancy microbial mediators in the pathway from maternal smoking during pregnancy to weight outcomes at 1 and 3 years.

We next investigated the effect of maternal smoking on the infant gut microbiota. According to the self-administered questionnaire, significant differences in the overall gut microbiota composition (measured by Bray-Curtis dissimilarity) were observed in both early infancy (*p* = .001) and late infancy (*p* = 0.025) between children who were or were not exposed to tobacco smoke during pregnancy through their mothers (Figure 2a). Alpha diversity indices of the gut microbiota in both early and late infancy were significantly increased in children with a history of exposure to maternal smoking during pregnancy (Table S7), particularly for all diversity indices of the Firmicutes phylum and the phylogenetic diversity index of Actinobacteria (Table S7, Figure 3a, Figure S5a).

(a) Principal coordinate analysis (PCoA) of beta diversity among the collected samples based on Bray-Curtis dissimilarity. Diagrams at the top side and on the right side represent the density of samples along the PCo1 and PCo2, respectively. *P* values were given by *adonis* tests. (b) Differentially abundant taxa identified by LEfSe. “Yes”: samples from infants whose mothers quit smoking during pregnancy, reduced the number of cigarettes, or had the same number of cigarettes (as before conception) during pregnancy. “No”: samples from infants whose mothers quit smoking before conception, or never smoked.

(a) Relative abundance or value of indices of the mediators by exposure of maternal smoking during pregnancy, year-1 weight status, and year-3 weight status. The category “wasted” was included here to show the trend, but not included in the downstream analysis because of its small sample size and being out of the scope of the study. *P* values: “***”, <0.001; “**”, <0.01; “*”, < 0.05; “.” < 0.1. *P* values in were FDR-corrected. (b) The proportions of the effect of maternal smoking during pregnancy on childhood weight outcome mediated through the microbial mediators. In the mediation analysis, maternal pre-pregnancy BMI, maternal race/ethnicity, prenatal and postnatal pet exposure, breastfeeding status at 3 months, solid food introduction at three months, and household disinfectant use, prenatal diet calories, infant sex, birth mode and antibiotic use in the first 3 months and age of sample collection (in months) were adjusted in the mediator (gut microbiota)-outcome associations, while maternal race/ethnicity and maternal education level was considered in the exposure (maternal smoking during pregnancy)-outcome association. The sizes of orange triangles reflected the mediation proportions. Significant mediation effects were marked in red.

As per the LEfSe analysis, several Firmicutes members, particularly those from the Clostridia class, were significantly enriched in children exposed to maternal smoking during pregnancy in early infancy (Figure 2b). These included Lachnospiraceae, Oscillospiraceae, *Ruminococcus gnavus*, *Ruminococcus torques*, and *Flavonifractor*. *Megasphaera* (a Veillonellaceae member) from the Firmicutes phylum and *Sutterella* from the Proteobacteria phylum were also higher in this group of children. In contrast, an increased abundance of *Blautia*, *Rothia*, and *Haemophilus* was observed in the non-exposed children (Figure 2b). In late infancy, we observed consistent directions of enrichment for Oscillospiraceae, *Ruminococcus torques*, *Flavonifractor*, *Sutterella*, and *Haemophilus* as were observed in early infancy. Additionally, increased abundance of Veillonellaceae, mainly *Veillonella*, was observed in the non-exposed group (Figure 2b). Notably, most of these differential taxa were significantly correlated with maternal smoking exposure levels during pregnancy (Table S8). Besides, in early infancy, their associations with maternal smoking during pregnancy were evident regardless of maternal pre-pregnancy weight status (Figure S5) or breastfeeding status at 3 months (Figure S6), with few exceptions observed for Lachnospiraceae and *Haemophilus*.

### Gut microbial features mediate the effect of maternal prenatal smoking on childhood BMI

We then identified potential gut microbiota mediators in the path from maternal smoking during pregnancy to childhood overweight and obesity. Among microbial features associated with maternal smoking, Chao1 richness and Faith’s phylogenetic diversity (Faith’s PD, a measure of alpha diversity incorporating phylogenetic distance between taxa) within Firmicutes, Lachnospiraceae, Oscillospiraceae, *Flavonifractor* and *Haemophilus* were significantly associated with BMI z-scores at both 1 and 3 years of age (*p* < 0.05, Figure 3a, Figure S7a). All these taxa and indices were related to Firmicutes and were positively associated with BMI z-scores, except *Haemophilus*, a member of the Proteobacteria phylum, which was inversely correlated with the outcomes.

These microbial features in early infancy significantly mediated the association between maternal smoking during pregnancy and childhood BMI z-scores, especially at the age of 3 years, although with smaller effect sizes (*p* < 0.05, Figure 3b). Among these mediators, the two Firmicutes diversity indices (Chao1 richness and Faith’s PD) mediated the highest proportions of the total effects, accounting for 23.3–24.6% of those on year-1 BMI z-scores, and 12.4–15.2% of those on year-3 BMI z-scores. Mediation by late-infancy microbiota was significant only for Firmicutes Chao1 richness (7.2%, *p* = 0.012) and Faith’s PD (7.1%, *p* = 0.024) (Figure S7b).

When we looked at mediation effects for the binary outcome (normal weight vs. AROW or OWOB), only the mediation effects of early-infancy *Haemophilus* abundance (8.9%, *p* = 0.020) and Firmicutes Chao1 richness (9.9%, *p* = 0.028) on weight status at age 3 years remained significant (Figure 3b, Figure S7b). Additionally, the early-infancy Firmicutes Faith’s phylogenetic diversity index, and abundances of Oscillospiraceae, Lachnospiraceae and *Haemophilus* collectively mediated 24.2% (95% CI 8.3% − 54.5%) of the effect on year-3 BMI z-scores and 18.6% (95% CI 3.2% − 41.7%) of the effect on the year-3 binary outcome (Table S9).

### Gut microbiota-derived butyrate is a potential metabolic mediator

**Figure 4.**
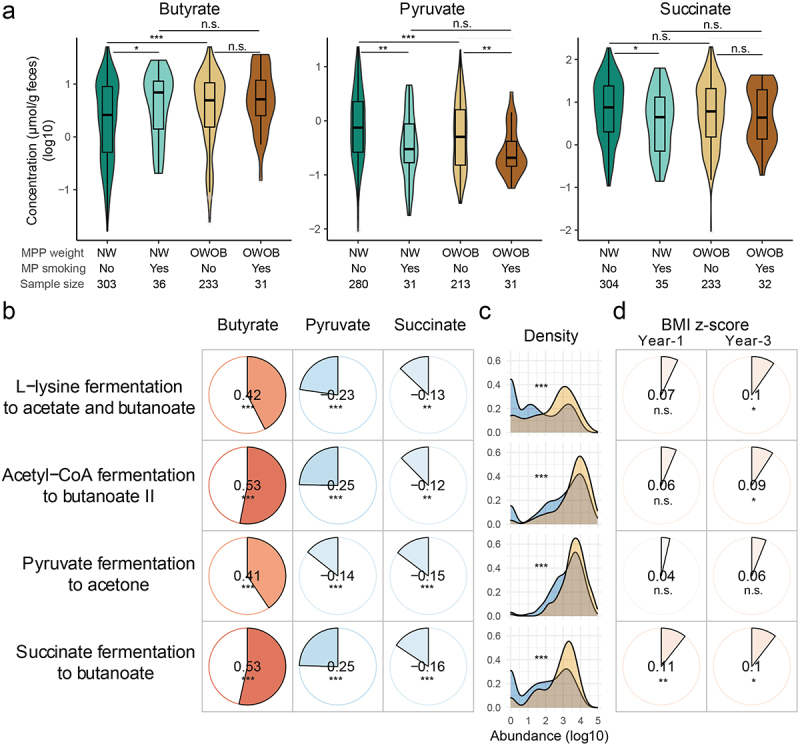
Metabolite mediators.

Since metabolites of the gut microbiota are known to be implicated in overweight and obesity, we further investigated metabolic mediators in these associations. Among early infancy fecal metabolites, we observed elevated butyrate but reduced pyruvate and succinate levels in the gut of children whose mothers had normal weight and smoked during pregnancy (Figure 4a); the concentrations of these metabolites were associated with maternal pre-pregnancy BMI (Table S10). In addition, partial or zero breastfeeding at 3 months was associated with higher butyrate levels across all smoking exposure strata (Figure S8a). Curiously, among exclusively breastfed infants, those whose mothers had quit smoking prior to pregnancy had the highest butyrate levels (Figure S8a). Moreover, butyrate was positively correlated with BMI z-scores at both age 1 (Spearman’s Rho = 0.13, *p* = .002) and 3 years (Spearman’s Rho = 0.14, *p* = .001), whereas pyruvate was inversely associated with BMI z-scores at 3 years of age (Spearman’s Rho = −0.16, *p* < .001) (Table S2). Interestingly, analysis of the PICRUSt2-predicted functional potential of early-infancy microbiota (Methods) showed that pathways linked to butyrate production, such as pathways for succinate fermentation to butyrate, L-lysine fermentation to acetate and butyrate, and acetyl-CoA fermentation to butyrate II, were higher in children with maternal smoke exposure during pregnancy and were significantly correlated with fecal butyrate, pyruvate, and succinate levels (*p* < .05, Figure 4b, c). These pathways were also positively associated with BMI z-scores at 3 years of age (*p* < .05), among which the pathway for succinate fermentation to butyrate was also significantly positively correlated with BMI z-scores at 1 year of age (*p* < .05, Figure 4d). In addition, the pathway for pyruvate fermentation to acetone was also elevated in children exposed to maternal smoke (*p* < .05; Figure 4b,c). These pathways were mainly contributed by *Flavonifractor* and *Lachnoclostridium* - two genera associated with exposure to maternal tobacco smoke during pregnancy, and *Veillonella*, *Bifidobacterium*, *Escherichia*/*Shigella*, *Bacteroides*, *Streptococcus*, and *Clostridium* sensu stricto (Cluster I) (Figure S8b). Among the predicted pathways of the late-infancy microbiota, the pathway for L-lysine fermentation to acetate and butyrate, and the pathway for acetyl-CoA fermentation to butyrate II were also increased in children with maternal smoke exposure (*p* < .05), but were not correlated with their BMI outcomes (*p* > .05). Finally, within a limited sample size with both fecal butyrate and weight outcome data available (*n* = 623), butyrate showed a trend to mediate the association between maternal prenatal smoking and certain childhood weight outcomes (proportion mediated in unadjusted models: year-1 BMI z-scores, 6.7%, *p* = .066; year-3 AROW or OWOB, 6.4%, *p* = .054; proportion mediated in adjusted models: year-1 BMI z-scores, 6.4%, *p* = .182; year-3 AROW or OWOB, 5.1%, *p* = .198).

(a) Fecal concentration of butyrate, pyruvate, and succinate in early infancy by maternal pre-pregnancy weight status and maternal smoking during pregnancy. (b) Correlations between butyrate, pyruvate, and succinate and related microbial pathways. c, Distributions of selected microbial pathways by maternal smoking during pregnancy. (d) Correlations between the selected microbial pathways and BMI z-scores at 1 and 3 years. *P* values: “***”, <0.001; “**”, <0.01; “*”, < 0.05; “.” < 0.1. *P* values in B, C, D were FDR-corrected.

## Discussion

In this large cohort study using a multi-omics approach, we are the first to identify several gut microbial mediators, mostly Firmicutes, of the effect of maternal smoking during pregnancy on childhood overweight and obesity, as well as potential metabolic players in these pathways. In general, Firmicutes are highly populated in obese subjects and have been positively associated with smoking in adults.^9,10^ Here, we build upon these findings and provide evidence of increased Firmicutes diversity in smoking mothers or children with maternal smoke exposure and its obesogenic role.

Being the main producers of short-chain fatty acids (SCFAs) like butyrate,^23^ higher Firmicutes diversity may result in increased butyrate production in the gut. In agreement with this hypothesis, we observed an elevation in fecal butyrate levels in children with a history of maternal smoking during pregnancy. On the other hand, the decreased concentration of pyruvate, an antagonist of monocarboxylate transporters involved in butyrate transport, may promote butyrate absorption by the host. Despite being generally considered beneficial for weight control,^24^ several cross-sectional studies have shown that obese subjects have higher fecal SCFA levels than lean ones.^25–27^ A recent study also showed a positive association between fecal butyrate concentration and child BMI z-scores in toddlers.^25^ In a clinical trial for weight control, the gut microbial capacity for butyrate production was found to be significantly reduced after a low-energy diet intervention.^28^ Another recent study based on the CHILD cohort reported the first piece of evidence for a temporal relationship between higher butyrate concentrations measured in early infancy and higher year-1 and year-3 BMI z-scores.^29^ The current study further illustrates that this relationship is partially attributed to exposure to maternal smoking during pregnancy, an effect that might be alleviated by maintaining exclusive breastfeeding for the first three months.

One possible mechanism of the positive butyrate-childhood obesity correlation is that an overabundance of available butyrate may result in the generation of excessive substrates for the citric acid cycle and lipid biosynthesis,^30^ which leads to extra weight gain. Additionally, butyrate can enhance growth hormone secretion by anterior pituitary cells in rats.^31^ This growth hormone increases energy expenditure and reduces fat mass in adults.^32^ Nonetheless, it also promotes weight gain in normal weight and underweight children.^33^

Taxa enriched in children with maternal tobacco smoke exposure, such as *Sutterella*, *Ruminococcus*, and Lachnospiraceae, were previously reported to be enriched in 12-month or mother’s samples compared to newborn or 4-month samples. Conversely, *Haemophilus*, which was depleted in children exposed to maternal smoking during pregnancy, was enriched in the newborns.^34^ Changes in the relative abundances of these age-related taxa, together with elevated Firmicutes diversity, indicate early maturation in the gut microbiota of children with maternal smoke exposure during pregnancy. In the first study quantifying gut microbiota maturity, Subramanian *et al*. reported persistently immature gut microbiota in malnourished children, which was transiently improved following dietary interventions.^35^ While the positive association between gut maturity and nutritional status in malnutrition cannot be directly extrapolated to the domain of obesity, our findings indicate that the acceleration of gut microbiota maturity in infancy may induce extra weight gain in childhood. This hypothesis is supported by repeated observations that partially or exclusively formula-fed infants have higher gut microbiota maturity,^36^ and are more likely to gain weight rapidly.^37^ These microbiota maturity related taxa, together with those relevant to SCFA production, may serve as potential predictive markers and intervention targets for childhood obesity regardless of smoke exposure during pregnancy.

Among other microbes associated with exposure to maternal smoking and/or childhood weight status, *Flavonifractor*^38^ and Lachnospiraceae^39^ were associated with increased inflammatory cytokines that may facilitate weight gain. *Haemophilus* was found to be more abundant in the oral microbiome of normal weight subjects and in nonsmokers than in obese and smokers,^40^ but was reported to be more abundant in obese subjects in another cohort.^41^ In contrast, *Flavonifractor plautti*, *R. torques*, and *R. gnavus* increased in abundance, whereas *Haemophilus parainfluenzae* decreased in abundance in adults with active or passive exposure to tobacco smoke in a Dutch cohort.^42^ *H. parainfluenzae* was also negatively associated with childhood smoke exposure.^42^ *Flanonifractor* and *Lachnoclostridium* were also enriched in children exposed to maternal smoking during pregnancy and were shown to mediate the associations of the Energy-Adjusted Dietary Inflammatory Index (E-DII score) with visceral adipose tissue and liver fat.^43^ Although further mechanistic investigation of these mediators is required, suppressing the disease-associated taxa (such as via phage therapy) may reduce the risk of developing obesity and relevant health conditions.

Our study also reports important observational evidence that quitting smoking or reducing the number of cigarettes smoked after conception does not lower the risk of childhood overweight and obesity.^44^ Smoking may also have a long-term and trans-generational effect through reprogramming gut microbiota and physiology, as higher butyrate levels were observed in the offspring of those who quit smoking prior to pregnancy than in those of nonsmokers. However, quitting smoking before conception can reduce the risk of childhood overweight and obesity. These findings add to the large body of evidence regarding the harmful effects of maternal smoking.^7,8^ The addition of these findings on obesity risk supplements public health practitioners’ call for potential policy interventions, parents’ decisions to quit maternal smoking prior to pregnancy, and nonsmokers’ decisions to try smoking.

This study was limited by the lack of gut microbiota samples from mothers. Whether maternal smoking during pregnancy affects unnecessary weight gain in children via vertical transmission of obesogenic gut microbiota warrants future longitudinal studies involving the collection of maternal gut microbiota samples. In addition, epigenetic alterations induced by smoke exposure, such as those in the aryl hydrocarbon receptor repressor (AhRR) gene, have been found to be transgenerational.^45,46^ The target of this repressor, AHR, is implicated in homeostasis^47^ and can interact bidirectionally with the gut microbiota bidirectionally.^48^ Therefore, exposure to maternal smoking during pregnancy may affect the assembly of gut microbiota in offspring through epigenetic effects. Furthermore, although we proposed a directed acyclic graph based on biological plausibility, the findings of the current study are still associated. Therefore, experimental studies are required to establish causality. Despite having a large cohort, we were unable to conduct further analyses for certain subgroups with a small sample size, such as testing the mediation role of breastfeeding in the causal pathway. Adjusting for this potential mediator as a covariate might have resulted in bias in the effect estimates. Lastly, a large-scale external cohort is warranted for validation but is currently not available.

In conclusion, this study revealed that the increased diversity of Firmicutes members mediates the effect of maternal smoking during pregnancy on childhood obesity in the offspring. This increase in the diversity of Firmicutes may play an obesogenic role via enhanced butyrate production, despite its general anti-obesity activities in adults. These findings highlight the mediating role of gut microbiota in the effect of prenatal detrimental exposure on childhood outcomes and open up avenues for the future development of microbiome-based weight control in pediatric populations.

## Methods

### Study design

To understand the influential factors of asthma, allergies, obesity, and other chronic diseases, the CHILD Cohort Study originally recruited 3,264 mothers during the third trimester of their pregnancies from four study sites between 2009 and 2012. Enrollment was confirmed upon singleton live birth at ≥35 weeks of gestation with a birth weight of ≥2,500 g. Major exclusion criteria were 1) *in vitro* fertilized births to avoid multiple gestation or preterm births, and 2) home births due to missing documentation of maternal intrapartum antibiotic prophylaxis. Mothers were followed up throughout pregnancy, and their children were assessed from birth to 3 years of age. The present study involved a large subset of 1,592 subjects with complete information on maternal smoking status during pregnancy, weight, length/height (at age 1 year and 3 years), and microbiota data (at approximately 3 months and 12 months of age). Samples from the Toronto site were excluded due to the lack of a provincial prescription database for assessing antibiotic medication.

The CHILD Cohort Study received initial approval from the Human Research Ethics Boards at the University of Manitoba, University of Alberta, and University of British Columbia. Written informed consent was obtained from the legal guardians of each participant. The present study was conducted as a sub-study program within the CHILD study, specifically approved by the University of Alberta (SyMBIOTA, Study ID: Pro00010073). Access to the data from the CHILD Cohort Study and the proposal for publication were both granted approval by CHILD (Concept Title C18, Concept Proposal #46, and Publication Proposal #1107).

### Exposure and outcome measurements

Maternal smoking status during pregnancy and covariates were collected through standardized questionnaires completed or obtained from hospital records by CHILD study staff (Supplementary Methods). Maternal smoking status during pregnancy was categorized into 1) never smoker, 2) quit smoking before conception, 3) quit smoking during pregnancy, 4) cut the number of cigarettes during pregnancy, and 5) had the same number of cigarettes during pregnancy. In modeling of the effect of detailed maternal smoking statuses during pregnancy, cutting the number of cigarettes during pregnancy and having the same number of cigarettes during pregnancy were combined into one category (“Yes”) due to small sample sizes. These detailed statuses were also dichotomized into 1) “Yes/Smoker”: those who quit smoking during pregnancy, reduced the number of cigarettes, or had the same number of cigarettes (as before conception) during pregnancy; and 2) “No/Non-smoker”: those who quit smoking before conception, or never smoked. Urine levels of two nicotine metabolites, cotinine and *trans*-3’- hydroxycotinine, measured at 3 months of age by liquid chromatography-atmospheric pressure chemical ionization tandem mass spectrometry (LC-MS), were used to verify the self-reported smoking status during pregnancy.

The weight and length/height of the children were measured by the CHILD study staff at 1 and 3 years of age. From these measures, sex-specific body mass index (BMI, kg/m^2^-for-age z-scores were computed according to WHO Child Growth reference data using the “igrowup” package for SPSS.^49^ Sex-specific BMI-for-age z-scores >5 or ≤ −5 were considered outliers and removed from downstream analyses. Weight status was categorized into “Wasted” (BMI z-score < −2), “Normal” (−2 ≤ BMI z-score ≤1), “At-risk overweight” (1 < BMI z-score ≤2), “Overweight” (2 < BMI z-score ≤3) and “Obese” (BMI z-score >3). Weight status was also dichotomized into “Normal” (−2 ≤ BMI z-score ≤1) and “AROW or OWOB” (BMI z-score >1), where those who were “Wasted” (BMI z-score < −2) were excluded.

### Taxonomic and metabolomic profiling of fecal microbiota

Fecal samples were collected in early infancy (at age 3.5 ± 1.0 months) during a planned home visit, and in late infancy (at age 12.3 ± 1.4 months) during a clinical study visit using a standard protocol.^50^ Methods of sample processing, DNA extraction, amplification, and sequencing (the V4 region of 16S ribosomal RNA) were described in our previous study.^18^ Amplicon sequence variants (ASVs) were generated from sequencing reads and annotated using the QIIME2 pipeline (v2020.6),^51^ based on which alpha indices (observed ASVs, Chao1 index, Shannon diversity, Simpson’s diversity and Faith’s phylogenetic diversity) and beta diversity (Bray-Curtis dissimilarity) were calculated, and metabolic functions were predicted using PICRUSt2 (Supplementary Methods).^52,53^

Fecal metabolites were measured in a subset of 3-month samples (*n* = 623) using magnetic resonance spectroscopy and quantified as micromoles per gram of feces (μmol/g).^54^ In this study, 33 metabolites detected in > 80% of the samples were analyzed. Nicotine metabolites, namely cotinine and 3-hydroxycotinine, were examined in a subset of samples (*n* = 1,368) collected at 3 months. Analysis was conducted using liquid chromatography-atmospheric pressure chemical ionization tandem mass spectrometry, and the results were reported as concentrations in ng/mL.^55^

### Statistical analysis

This study was conducted within the CHILD cohort using all available data, and a sample size calculation was not applicable. For bivariate analysis, the Fisher’s exact test was used for categorical data. T-tests/analysis of variance (ANOVA) and Wilcoxon’s tests/Kruskal-Wallis tests were performed for two/four-group comparisons of continuous parameters that were and were not normally distributed. Dunn’s test was used for pairwise comparisons of continuous data between multiple groups, and false discovery rate (FDR) correction was performed using the Benjamini-Hochberg method for multiple comparisons. Spearman’s and Pearson’s tests were performed for correlations between continuous data where appropriate.

Generalized linear models were used to estimate the effect sizes. When modeling the maternal smoking-BMI z-score/weight status relationship, potential confounders and covariates were adjusted for. In Model 1, we adjusted for 1) the minimum set of confounders identified by a direct acyclic graph (DAG) analysis, that is, maternal race/ethnicity and socioeconomic status, and 2) maternal pre-pregnancy BMI, a known factor correlating with childhood weight status (except in the models assessing the joint effects of maternal BMI and smoking status). In Model 2, we further controlled for variables differentially distributed between the exposed and non-exposed groups, including prenatal and postnatal pet exposure, breastfeeding status at three months (except in the models assessing the joint effects of maternal smoking status and breastfeeding status at three months), solid food introduction at three months, and household disinfectant use. Finally, in Model 3, we also controlled for factors that were potentially correlated with the BMI z-scores of children, including prenatal diet calories, infant sex, birth mode, and antibiotic use in the first three months. In addition, the *interactionR* of R package “interactionR”^56^ was used to investigate potential interactions between maternal smoking during pregnancy and maternal pre-pregnancy weight status/breast feeding status, with covariates controlled for as in Model 3 above. Maternal education level was adjusted as a proxy for socioeconomic status. Differentially abundant microbial taxa between the two groups were identified using linear discriminant analysis effect size (LEfSe).^57^

Mediation analysis was conducted using the *mediate* function of R package “mediation”,^58^ while confidence intervals and *p* values were generated using 1,000 bootstraps. To account for confounding effects of exposures and life-style factors on BMI outcomes and microbial abundance or alpha diversity indices, propensity scores were first estimated by the Covariate Balancing Propensity Score (*CBPS*) method implemented in R package “CBPS”.^59^ According to the DAG (Figure S3), the fitted factors included maternal race/ethnicity, maternal pre-pregnancy BMI, prenatal and postnatal pet exposure, breastfeeding status at three months, solid food introduction at three months, household disinfectant use, prenatal diet calories, infant sex, birth mode, antibiotic use and the age of sample collection. The propensity score, together with socioeconomic status and maternal race/ethnicity, were then included in the mediation analysis. In addition, the *mma* function of R package “Multiple Mediation Analysis (mma)”^60^ was used to explore the combined mediation effects of the potential mediators while adjusting for the covariates mentioned above. In this analysis, Firmicutes Faith’s phylogenetic diversity index, Oscillospiraceae, Lachnospiraceae and *Haemophilus* were included. Firmicutes Chao1 index was not included because it was highly correlated with the Firmicutes Faith’s phylogenetic diversity index; while *Flavonifractor* was not included as its family Oscillospiraceae has a larger effect size in the separate model.

In differential abundance analysis, correlation analysis and modeling, microbial taxa (phylum to genus) with a prevalence of < 1% or a mean abundance of < 0.1% at each time point were removed; relative abundance and alpha diversity indices were subjected to rank-based inverse normal transformation.^61^

Pairwise deletion was performed in bivariate analysis, whereas listwise deletion was performed in modeling missing data. Statistical significance was set at *p* = 0.05. All statistical analyses were performed using R version 4.1.0.

## Supplementary Material

R2_Supplementary_materials_clean.docx

## Data Availability

De-identified data are available from the corresponding author upon reasonable request. Registration and/or proposal applications may be needed depending on the tier of access requested (https://childstudy.ca/for-researchers/data-access/).
