# Supplementary material for: Maternal smoking during pregnancy increases the risk of gut microbiome-associated childhood overweight and obesity
Source: Gut Microbes. 2024 Mar 4;16(1):2323234. doi: 10.1080/19490976.2024.2323234 (PMC10913716; doi:10.1080/19490976.2024.2323234)
Supplement: R2_Supplementary_materials_clean.docx [file KGMI_A_2323234_SM0287.docx]

Supplementary Materials

Table of Contents

[Supplementary Methods 2](#_Toc144850480)

[Exposure and covariate measurements 2](#_Toc144850481)

[Fecal microbiota analysis 2](#_Toc144850482)

[Supplementary Tables 4](#_Toc144850483)

[Supplementary Figures 23](#_Toc144850484)

[Supplementary References 29](#_Toc144850485)

# Supplementary Methods

## Exposure and covariate measurements

Status on maternal smoking during pregnancy and covariates were collected through standardized questionnaires completed by mothers [maternal smoking during pregnancy, maternal BMI, maternal race/ethnicity, maternal education level, breastfeeding status, presence of adults, siblings, and furry pets at home, infant antibiotic exposure, dietary habits (solid food introduction, vitamin supplements, having juice, use of plastic bottles, and food frequency at year 3), and household disinfectant use] or obtained from hospital records [mode of delivery, intrapartum antibiotic prophylaxis (IAP)].

## Fecal microbiota analysis

Amplicon sequence variants (ASVs) were generated from sequencing reads and annotated using the QIIME2 pipeline (v2020.6) ^1^. Briefly, forward and reverse sequencing reads were trimmed per the base-quality distribution into 150bp and 134bp, respectively, and then assembled into amplicon sequence variants (ASVs) by DADA2. Unique ASVs were assigned a taxonomy by a classifier (the classify-sklearn method from the q2-feature-classifier plugin) trained on the SILVA reference database (Release 138, at 99% sequence identity). Data were rarefied to 49,000 sequences per sample to adjust the various sequencing depths among samples. The relative abundance of the annotated ASVs was summarized at the phylum to genus levels. Microbial alpha diversity within samples was calculated as Chao1 richness, observed ASVs, Faith’s phylogenetic diversity (PD) whole tree index (a measure of alpha diversity incorporating phylogenetic difference between taxa), Simpson index, and Shannon index based on ASV profiles. These alpha diversity indices were presented for the whole microbiota as well as under each specific phylum of four dominant phyla (Actinobacteria, Bacteroidetes, Firmicutes, and Proteobacteria) in infant gut. Microbial community differences between samples (beta diversity) were measured as Bray-Curtis dissimilarity between the ASV profiles. Metagenomic functions were predicted from the ASV table using the PICRUSt2 (phylogenetic investigation of communities by reconstruction of unobserved states) pipeline ^2^ based on the MetaCyc database ^3^.

# Supplementary Tables

Table S1. Child food frequency at 3 years by weight status.

|  | Year-3 AROW | | | |
| --- | --- | --- | --- | --- |
| Food item | NW | AROW | *P* value | FDR |
| Milk | 7 (6, 7) | 7 (6, 7) | 0.066 | 0.439 |
| Hot Chocolate | 0 (0, 1) | 0 (0, 1) | 0.78 | 0.922 |
| Milk Shake | 0 (0, 0) | 0 (0, 0) | 0.66 | 0.903 |
| Cheese | 4 (4, 6) | 5 (4, 6) | 0.009 | 0.35 |
| Cheese | 0 (0, 0) | 0 (0, 0) | 0.069 | 0.439 |
| Cheese Spread | 0 (0, 0) | 0 (0, 0) | 0.909 | 0.971 |
| Cream Cheese | 0 (0, 1) | 0 (0, 1) | 0.35 | 0.745 |
| Yogurt | 4 (3, 6) | 4 (4, 6) | 0.406 | 0.765 |
| Mini Go | 0 (0, 1) | 0 (0, 1) | 0.532 | 0.818 |
| Ice Cream | 2 (1, 3) | 2 (1, 3) | 0.511 | 0.808 |
| Yogurt Drink | 0 (0, 1) | 0 (0, 1) | 0.987 | 0.99 |
| Soy Drink | 0 (0, 0) | 0 (0, 0) | 0.024 | 0.35 |
| Rice Drink | 0 (0, 0) | 0 (0, 0) | 0.621 | 0.897 |
| Butter | 3 (0.25, 4) | 3 (0, 4) | 0.29 | 0.704 |
| Margarine | 0 (0, 4) | 1 (0, 4) | 0.022 | 0.35 |
| Banana | 4 (3, 4) | 4 (3, 5) | 0.006 | 0.35 |
| Peaches | 0 (0, 2) | 1 (0, 2) | 0.365 | 0.751 |
| Fruit cocktail, mixed fruit | 0 (0, 1) | 0 (0, 1) | 0.425 | 0.786 |
| Orange | 3 (1, 4) | 3 (2, 4) | 0.695 | 0.903 |
| Grapefruit | 0 (0, 0) | 0 (0, 0) | 0.518 | 0.808 |
| Apple | 4 (3, 5) | 4 (3, 5) | 0.39 | 0.765 |
| Pear | 2 (0, 3) | 1 (0, 3) | 0.276 | 0.688 |
| Grapes | 3 (2, 4) | 3 (2, 4) | 0.306 | 0.71 |
| Strawberries | 3 (2, 4) | 3 (2, 4) | 0.229 | 0.639 |
| Watermelon | 1 (0, 3) | 1 (0, 3) | 0.295 | 0.704 |
| Cantaloupe | 1 (0, 2) | 1 (0, 2) | 0.271 | 0.688 |
| Pineapple | 1 (0, 2) | 1 (0, 2) | 0.469 | 0.808 |
| Applesauce | 1 (0, 3) | 1 (0, 3) | 0.97 | 0.987 |
| Dried fruits | 1 (0, 3) | 1 (0, 3) | 0.151 | 0.519 |
| Sorbet | 0 (0, 0) | 0 (0, 0) | 0.921 | 0.971 |
| Orange juice/ grapefruit juice | 1 (0, 3) | 1 (0, 3) | 0.736 | 0.906 |
| Other juice | 3 (1, 4) | 3 (1, 5) | 0.087 | 0.482 |
| Corn | 2 (1, 3) | 2 (1, 3) | 0.332 | 0.732 |
| Peas | 2 (0.25, 3) | 2 (1, 3) | 0.145 | 0.519 |
| Tomatoes | 3 (0, 4) | 3 (0.25, 4) | 0.274 | 0.688 |
| Peppers | 2 (0, 4) | 2 (0, 4) | 0.22 | 0.627 |
| Carrots | 4 (3, 4) | 4 (3, 4) | 0.197 | 0.62 |
| Broccoli | 3 (2, 4) | 3 (1, 4) | 0.042 | 0.389 |
| Green beans | 1 (0, 3) | 1 (0, 3) | 0.803 | 0.94 |
| Spinach | 0 (0, 2) | 1 (0, 2) | 0.478 | 0.808 |
| Greens | 0 (0, 2) | 0 (0, 1) | 0.095 | 0.482 |
| Mixed vegetables | 2 (0, 4) | 2 (0, 4) | 0.398 | 0.765 |
| Squash, orange or winter | 0 (0, 1) | 0 (0, 1) | 0.743 | 0.906 |
| Zucchini, yellow squash | 0 (0, 2) | 0 (0, 2) | 0.688 | 0.903 |
| Cabbage, coleslaw | 0 (0, 1) | 0 (0, 1) | 0.494 | 0.808 |
| Lettuce salad | 1 (0, 3) | 1 (0, 3) | 0.313 | 0.71 |
| Cauliflower | 1 (0, 3) | 1 (0, 2) | 0.736 | 0.906 |
| Tofu | 0 (0, 1) | 0 (0, 0) | 0.017 | 0.35 |
| Cold cereal | 4 (3, 5) | 4 (3, 5) | 0.668 | 0.903 |
| Hot cereal, grits | 2 (0, 4) | 2 (0, 4) | 0.849 | 0.951 |
| Bread - pita | 0 (0, 2) | 0 (0, 2) | 0.845 | 0.951 |
| Bread | 4 (4, 6) | 5 (4, 6) | 0.039 | 0.389 |
| English muffin | 0 (0, 1) | 0 (0, 1) | 0.709 | 0.903 |
| Bagel | 0 (0, 2) | 0 (0, 2) | 0.854 | 0.951 |
| Waffles | 0 (0, 2) | 0 (0, 2) | 0.124 | 0.51 |
| Sweet Roll or muffin | 1 (0, 2) | 1 (0, 2) | 0.705 | 0.903 |
| Tacos, burritos | 0 (0, 2) | 0 (0, 2) | 0.765 | 0.922 |
| Pancakes | 2 (1, 3) | 2 (1, 3) | 0.339 | 0.735 |
| French toast | 0 (0, 1) | 0 (0, 1) | 0.437 | 0.786 |
| Egg McMuffin | 0 (0, 0) | 0 (0, 0) | 0.641 | 0.903 |
| Macaroni & cheese | 2 (1, 3) | 2 (1, 3) | 0.071 | 0.439 |
| Spaghetti or other pasta | 3 (2, 4) | 3 (2, 4) | 0.678 | 0.903 |
| Rice | 3 (2, 4) | 3 (2, 4) | 0.126 | 0.51 |
| Pizza | 2 (1, 3) | 2 (1, 3) | 0.054 | 0.418 |
| Lasagna | 1 (0, 1) | 1 (0, 1) | 0.211 | 0.62 |
| Potatoes | 2 (1, 3) | 2 (1, 3) | 0.114 | 0.51 |
| French fries, fried potatoes, tater tots | 2 (1, 3) | 2 (1, 3) | 0.094 | 0.482 |
| Sweet potatoes or yams | 1 (0, 2) | 1 (0, 2) | 0.374 | 0.754 |
| Granola bar | 2 (0, 4) | 2 (0, 4) | 0.586 | 0.879 |
| Pie | 0 (0, 1) | 0 (0, 1) | 0.99 | 0.99 |
| Doughnut | 0 (0, 1) | 1 (0, 1) | 0.616 | 0.897 |
| Crackers | 4 (3, 4) | 4 (3, 5) | 0.108 | 0.51 |
| Corn bread or tortilla | 0 (0, 2) | 0 (0, 2) | 0.603 | 0.893 |
| Cookies or brownies | 3 (2, 4) | 3 (2, 4) | 0.709 | 0.903 |
| Cake or cupcake | 1 (1, 2) | 1 (1, 2) | 0.433 | 0.786 |
| Tea Biscuit | 0 (0, 0) | 0 (0, 0) | 0.366 | 0.751 |
| Popcorn or pretzels | 1 (0, 2) | 2 (1, 3) | 0.025 | 0.35 |
| Beans | 1 (0, 2) | 1 (0, 2) | 0.557 | 0.846 |
| Lentils | 0 (0, 1) | 0 (0, 1) | 0.13 | 0.51 |
| Eggs | 4 (2, 4) | 4 (2, 4) | 0.136 | 0.512 |
| Luncheon meat | 1 (0, 3) | 2 (0, 3) | 0.131 | 0.51 |
| Pork or ham | 2 (1, 3) | 2 (1, 3) | 0.639 | 0.903 |
| Roast beef or steak | 2 (0, 3) | 2 (0, 3) | 0.162 | 0.541 |
| Weiner | 1 (0, 2) | 2 (0, 3) | 0.171 | 0.555 |
| Sausage | 1 (0, 2) | 1 (0, 2.75) | 0.027 | 0.35 |
| Hamburger | 2 (1, 3) | 2 (1, 3) | 0.018 | 0.35 |
| Bacon | 1 (0, 2) | 1 (0, 2) | 0.479 | 0.808 |
| Meatballs | 1 (0, 2) | 1 (0, 2) | 0.921 | 0.971 |
| Cold cuts | 1 (0, 2) | 1 (0, 2) | 0.739 | 0.906 |
| Stir fried chicken | 1 (0, 2) | 1 (0, 2) | 0.862 | 0.951 |
| Fried chicken, chicken nuggets | 1 (0, 2) | 1 (0, 2) | 0.212 | 0.62 |
| Other chicken or turkey | 3 (1, 4) | 3 (2, 4) | 0.874 | 0.956 |
| Shrimp | 0 (0, 1) | 0 (0, 1) | 0.843 | 0.951 |
| Canned tuna | 0 (0, 1) | 0 (0, 1) | 0.038 | 0.389 |
| Fried fish, fish sticks | 0 (0, 1) | 0 (0, 1) | 0.067 | 0.439 |
| Other fish | 1 (0, 2) | 1 (0, 2) | 0.087 | 0.482 |
| Vegetable soup | 1 (0, 2) | 1 (0, 2) | 0.046 | 0.389 |
| Other soup | 2 (0, 3) | 2 (0, 3) | 0.889 | 0.963 |
| Liver, organ meats | 0 (0, 0) | 0 (0, 0) | 0.514 | 0.808 |
| Nuts | 2 (1, 4) | 2 (0, 3) | 0.149 | 0.519 |
| Peanut butter | 3 (1, 4) | 3 (2, 4) | 0.208 | 0.62 |
| Salad dressing | 1 (0, 3) | 1 (0, 3) | 0.71 | 0.903 |
| Mayonnaise | 1 (0, 2) | 1 (0, 2) | 0.933 | 0.975 |
| Chocolate or candy bar | 2 (1, 3) | 2 (1, 3) | 0.777 | 0.922 |
| Other candy, not chocolate | 2 (1, 3) | 2 (1, 3) | 0.267 | 0.688 |
| Fruit drinks | 1 (0, 3) | 2 (0, 4) | 0.275 | 0.688 |
| Soda, soft drinks, pop (regular) | 0 (0, 1) | 0 (0, 1) | 0.853 | 0.951 |
| Soda, soft drinks, pop (sugar free) | 0 (0, 0) | 0 (0, 0) | 0.047 | 0.389 |
| Jello | 0 (0, 1) | 0 (0, 1) | 0.5 | 0.808 |
| Pudding | 0 (0, 1) | 0 (0, 1) | 0.009 | 0.35 |
| Potato Chips | 1 (0, 2) | 1 (0, 2) | 0.97 | 0.987 |
| Corn Chips (nachos) | 1 (0, 2) | 1 (0, 2) | 0.505 | 0.808 |
| Multivitamin | 1 (0, 2) | 1 (0, 2) | 0.115 | 0.51 |
| Iron Supplement | 2 (2, 2) | 2 (2, 2) | 0.965 | 0.987 |
| Vitamin A Supplement | 2 (2, 2) | 2 (2, 2) | 0.47 | 0.808 |
| Calcium Supplement | 2 (2, 2) | 2 (2, 2) | 0.316 | 0.71 |
| Vitamin D Supplement | 2 (1, 2) | 2 (1, 2) | 0.4 | 0.765 |

*P* values were given by Wilcoxon’s rank-sum test.

Table S2. Associations between subject characteristics and weight outcomes at 1 and 3 years.

|  | **Year 1 BMI z-score** | | |  | **Year 3 BMI z-score** | | |
| --- | --- | --- | --- | --- | --- | --- | --- |
| **Continuous variables** | **N** | **Rho** | ***P* value** |  | **N** | **Rho** | ***P* value** |
| Maternal pre-pregnancy BMI | 1549 | 0.14 | <0.001 |  | 1410 | 0.19 | <0.001 |
| Maternal education | 1571 | -0.09 | <0.001 |  | 1424 | -0.1 | <0.001 |
| Total calories of maternal prenatal diet | 1518 | 0.04 | 0.084 |  | 1376 | 0.07 | 0.014 |
| Urinary Cotinine concentration (log2) | 1368 | 0.09 | <0.001 |  | 1238 | 0.1 | <0.001 |
| Urinary Hydroxycotinine concentration (log2) | 1368 | 0.08 | 0.002 |  | 1238 | 0.13 | <0.001 |
|  |  |  |  |  |  |  |  |
| **Categorical variables** | N (%) | Mean (s.d.) | P value |  | N (%) | Mean (s.d.) | P value |
| Maternal smoking during pregnancy | 1592 |  | <0.001 |  | 1441 |  | <0.001 |
| Never | 1151 (72.3) | 0.14 (1.07) |  |  | 1050 (72.9) | 0.57 (0.97) |  |
| Quit prior to pregnancy | 307 (19.3) | 0.2 (1.05) |  |  | 279 (19.4) | 0.62 (1.03) |  |
| Quit during pregnancy | 68 (4.3) | 0.45 (0.89) |  |  | 59 (4.1) | 0.95 (0.88) |  |
| Yes, cut the number | 54 (3.4) | 0.54 (1.11) |  |  | 45 (3.1) | 0.97 (0.79) |  |
| Yes, the same number | 12 (0.8) | 1.02 (0.79) |  |  | 8 (0.6) | 2.04 (1.35) |  |
| Maternal smoking during pregnancy | 1592 |  | <0.001 |  | 1441 |  | <0.001 |
| Never | 1151 (72.3) | 0.14 (1.07) |  |  | 1050 (72.9) | 0.57 (0.97) |  |
| Quit prior to pregnancy | 307 (19.3) | 0.2 (1.05) |  |  | 279 (19.4) | 0.62 (1.03) |  |
| Quit during pregnancy | 68 (4.3) | 0.45 (0.89) |  |  | 59 (4.1) | 0.95 (0.88) |  |
| Yes | 66 (4.1) | 0.63 (1.07) |  |  | 53 (3.7) | 1.13 (0.96) |  |
| Maternal smoking during pregnancy | 1592 |  | <0.001 |  | 1441 |  | <0.001 |
| No | 1458 (91.6) | 0.15 (1.06) |  |  | 1329 (92.2) | 0.58 (0.98) |  |
| Yes | 134 (8.4) | 0.54 (0.98) |  |  | 112 (7.8) | 1.04 (0.92) |  |
| Maternal race | 1590 |  | <0.001 |  | 1439 |  | <0.001 |
| Asian | 236 (14.8) | 0 (1.06) |  |  | 217 (15.1) | 0.4 (0.92) |  |
| Caucasian | 1205 (75.8) | 0.19 (1.05) |  |  | 1092 (75.9) | 0.63 (0.98) |  |
| Other | 149 (9.4) | 0.43 (1.16) |  |  | 130 (9) | 0.86 (1.04) |  |
| Breast feeding at 3M | 1589 |  | <0.001 |  | 1438 |  | <0.001 |
| Exclusive BF | 904 (56.9) | 0.05 (1.04) |  |  | 831 (57.8) | 0.52 (0.96) |  |
| Partial BF | 429 (27) | 0.29 (1.05) |  |  | 378 (26.3) | 0.73 (0.95) |  |
| Formula | 256 (16.1) | 0.48 (1.09) |  |  | 229 (15.9) | 0.77 (1.09) |  |
| Pet exposure | 1529 |  | 0.005 |  | 1384 |  | 0.011 |
| No | 709 (46.4) | 0.12 (1.09) |  |  | 635 (45.9) | 0.53 (1.03) |  |
| Both Pre and Postnatal | 686 (44.9) | 0.29 (1.01) |  |  | 629 (45.4) | 0.71 (0.94) |  |
| Only Postnatal | 16 (1) | -0.3 (0.98) |  |  | 14 (1) | 0.59 (1.05) |  |
| Only prenatal | 118 (7.7) | 0.15 (0.98) |  |  | 106 (7.7) | 0.57 (0.97) |  |
| Plastic bottle sippy cup (3M) | 1342 |  | <0.001 |  | 1214 |  | 0.053 |
| 0 | 479 (35.7) | 0.01 (1.07) |  |  | 430 (35.4) | 0.59 (0.98) |  |
| 1 | 279 (20.8) | 0.04 (1.01) |  |  | 257 (21.2) | 0.54 (0.88) |  |
| 2 | 220 (16.4) | 0.21 (0.94) |  |  | 203 (16.7) | 0.59 (1.03) |  |
| 3 | 364 (27.1) | 0.44 (1.1) |  |  | 324 (26.7) | 0.75 (1.04) |  |
| Juice (3M) | 1340 |  | 0.025 |  | 1212 |  | 0.496 |
| No | 1320 (98.5) | 0.16 (1.06) |  |  | 1193 (98.4) | 0.62 (0.98) |  |
| Yes | 20 (1.5) | 0.69 (1.14) |  |  | 19 (1.6) | 0.77 (1.53) |  |
| Vitamins or other supplements (3M) | 1304 |  | 0.731 |  | 1184 |  | 0.972 |
| No | 440 (33.7) | 0.18 (1.13) |  |  | 403 (34) | 0.61 (1.02) |  |
| Yes | 864 (66.3) | 0.16 (1.01) |  |  | 781 (66) | 0.62 (0.96) |  |
| Solid food introduction at 3M | 1580 |  | 0.482 |  | 1431 |  | 0.788 |
| No | 1537 (97.3) | 0.18 (1.07) |  |  | 1392 (97.3) | 0.62 (0.98) |  |
| Yes | 43 (2.7) | 0.3 (0.98) |  |  | 39 (2.7) | 0.66 (1.04) |  |
| Birth mode | 1547 |  | 0.199 |  | 1403 |  | 0.322 |
| Vaginal_noIAP | 834 (53.9) | 0.14 (1.07) |  |  | 757 (54) | 0.58 (0.93) |  |
| Vaginal_IAP | 346 (22.4) | 0.24 (1.08) |  |  | 320 (22.8) | 0.7 (1.05) |  |
| CS_Elective | 149 (9.6) | 0.28 (1.05) |  |  | 129 (9.2) | 0.62 (1) |  |
| CS_Emergency | 218 (14.1) | 0.25 (1.02) |  |  | 197 (14) | 0.63 (1.09) |  |
| Direct use of antibiotic (first 3M) | 1481 |  | 0.469 |  | 1344 |  | 0.172 |
| No | 1353 (91.4) | 0.18 (1.07) |  |  | 1228 (91.4) | 0.61 (1) |  |
| Yes | 128 (8.6) | 0.25 (1.03) |  |  | 116 (8.6) | 0.74 (0.94) |  |
| Postnatanl disinfectant use | 1549 |  | 0.245 |  | 1404 |  | 0.059 |
| No | 724 (46.7) | 0.16 (1.03) |  |  | 658 (46.9) | 0.56 (0.98) |  |
| Yes | 825 (53.3) | 0.22 (1.08) |  |  | 746 (53.1) | 0.66 (1) |  |
| Number of adults at home | 1569 |  | 0.327 |  | 1419 |  | 0.34 |
| 1 | 33 (2.1) | 0.18 (1.26) |  |  | 27 (1.9) | 0.87 (1.04) |  |
| 2 | 1274 (81.2) | 0.21 (1.06) |  |  | 1158 (81.6) | 0.61 (1) |  |
| >=3 | 262 (16.7) | 0.1 (1.02) |  |  | 234 (16.5) | 0.64 (0.92) |  |
| Having siblings | 1567 |  | 0.465 |  | 1418 |  | 0.74 |
| Yes | 783 (50) | 0.17 (1.06) |  |  | 701 (49.4) | 0.61 (0.95) |  |
| No | 784 (50) | 0.21 (1.05) |  |  | 717 (50.6) | 0.63 (1.01) |  |
| Sex | 1592 |  | 0.544 |  | 1441 |  | 0.162 |
| Female | 741 (46.5) | 0.17 (1.02) |  |  | 668 (46.4) | 0.58 (0.92) |  |
| Male | 851 (53.5) | 0.2 (1.1) |  |  | 773 (53.6) | 0.65 (1.04) |  |

*P* values for comparisons of continuous data between groups were given by Wilcoxon’s tests or *t* tests where appropriate; *P* values for comparisons of categorical data between groups were given by Fisher’s exact tests.

Table S3. Interactions between maternal smoking during pregnancy and breastfeeding status in the associations with weight outcomes at 1 and 3 years.

|  | EBF |  | non-EBF |  | Effect of non-EBF within the strata of maternal smoking during pregnancy |  |
| --- | --- | --- | --- | --- | --- | --- |
| **Year-1 BMI** | OR (95% CI) | *P* value | OR (95% CI) | *P* value | OR (95% CI) | *P* value |
| Maternal smoking during pregnancy - No | 1 (Reference) | NA | 1.24 (1.09, 1.4) | **<0.001** | 1.24 (1.09, 1.4) | **<0.001** |
| Maternal smoking during pregnancy - Yes | 0.92 (0.63, 1.34) | 0.661 | 1.64 (1.26, 2.13) | **<0.001** | 1.79 (1.15, 2.77) | **0.009** |
| Effect of maternal smoking during pregnancy within the strata of breastfeeding at 3 months | 0.92 (0.63, 1.34) | 0.661 | 1.33 (1.03, 1.72) | **0.03** |  |  |
| Multiplicative scale | 1.45 (0.92, 2.27) | 0.109 |  |  |  |  |
| Additive scale (RERI) | 0.49 (-0.06, 1.02) | NA |  |  |  |  |
|  |  |  |  |  |  |  |
| **Year-3 BMI** |  |  |  |  |  |  |
| Maternal smoking during pregnancy - No | 1 (Reference) | NA | 1.14 (1.01, 1.28) | **0.034** | 1.14 (1.01, 1.28) | **0.034** |
| Maternal smoking during pregnancy - Yes | 1.22 (0.84, 1.76) | 0.299 | 1.54 (1.19, 2.01) | **0.001** | 1.27 (0.83, 1.95) | 0.277 |
| Effect of maternal smoking during pregnancy within the strata of breastfeeding at 3 months | 1.22 (0.84, 1.76) | 0.299 | 1.36 (1.05, 1.76) | **0.021** |  |  |
| Multiplicative scale | 1.12 (0.72, 1.74) | 0.624 |  |  |  |  |
| Additive scale (RERI) | 0.19 (-0.44, 0.75) | NA |  |  |  |  |
|  |  |  |  |  |  |  |
| **Year-1 AROW** |  |  |  |  |  |  |
| Maternal smoking during pregnancy - No | 1 (Reference) | NA | 1.46 (1.08, 1.96) | **0.012** | 1.46 (1.08, 1.96) | **0.012** |
| Maternal smoking during pregnancy - Yes | 0.82 (0.3, 2.25) | 0.707 | 2.01 (1.14, 3.55) | **0.016** | 2.44 (0.82, 7.25) | 0.109 |
| Effect of maternal smoking during pregnancy within the strata of breastfeeding at 3 months | 0.82 (0.3, 2.25) | 0.707 | 1.38 (0.79, 2.4) | 0.256 |  |  |
| Multiplicative scale | 1.67 (0.54, 5.14) | 0.369 |  |  |  |  |
| Additive scale (RERI) | 0.73 (-0.91, 2.24) | NA |  |  |  |  |
|  |  |  |  |  |  |  |
| **Year-3 AROW** |  |  |  |  |  |  |
| Maternal smoking during pregnancy - No | 1 (Reference) | NA | 1.13 (0.86, 1.49) | 0.377 | 1.13 (0.86, 1.49) | 0.377 |
| Maternal smoking during pregnancy - Yes | 1.45 (0.65, 3.23) | 0.368 | 2.21 (1.25, 3.9) | **0.006** | 1.53 (0.6, 3.86) | 0.379 |
| Effect of maternal smoking during pregnancy within the strata of breastfeeding at 3 months | 1.45 (0.65, 3.23) | 0.368 | 1.95 (1.11, 3.43) | **0.02** |  |  |
| Multiplicative scale | 1.35 (0.52, 3.53) | 0.541 |  |  |  |  |
| Additive scale (RERI) | 0.63 (-1.33, 2.39) | NA |  |  |  |  |

RERI: relative excess risk due to interaction.

Table S4. Interactions between maternal smoking during pregnancy and maternal pre-pregnancy weight status in the associations with weight outcomes at 1 and 3 years.

|  | mNW |  | mOWOB |  | Effect of mOWOB within the strata of maternal smoking during pregnancy |  |
| --- | --- | --- | --- | --- | --- | --- |
| **Year-1 BMI** | OR (95% CI) | *P* value | OR (95% CI) | *P* value | OR (95% CI) | *P* value |
| Maternal smoking during pregnancy - No | 1 (Reference) | NA | 1.22 (1.08, 1.38) | **0.002** | 1.22 (1.08, 1.38) | **0.002** |
| Maternal smoking during pregnancy - Yes | 1.28 (0.96, 1.71) | 0.091 | 1.27 (0.93, 1.75) | 0.136 | 0.99 (0.67, 1.47) | 0.976 |
| Effect of maternal smoking during pregnancy within the strata of maternal pre-pregnancy weight status | 1.28 (0.96, 1.71) | 0.091 | 1.04 (0.76, 1.43) | 0.807 |  |  |
| Multiplicative scale | 0.81 (0.54, 1.23) | 0.326 |  |  |  |  |
| Additive scale (RERI) | -0.23 (-0.77, 0.31) | NA |  |  |  |  |
|  |  |  |  |  |  |  |
| **Year-3 BMI** |  |  |  |  |  |  |
| Maternal smoking during pregnancy - No | 1 (Reference) | NA | 1.41 (1.25, 1.59) | **<0.001** | 1.41 (1.25, 1.59) | **<0.001** |
| Maternal smoking during pregnancy - Yes | 1.39 (1.06, 1.84) | **0.02** | 1.78 (1.29, 2.47) | **<0.001** | 1.28 (0.86, 1.91) | 0.227 |
| Effect of maternal smoking during pregnancy within the strata of maternal pre-pregnancy weight status | 1.39 (1.06, 1.84) | **0.02** | 1.27 (0.91, 1.75) | 0.156 |  |  |
| Multiplicative scale | 0.91 (0.6, 1.38) | 0.653 |  |  |  |  |
| Additive scale (RERI) | -0.02 (-0.67, 0.71) | NA |  |  |  |  |
|  |  |  |  |  |  |  |
| **Year-1 AROW** |  |  |  |  |  |  |
| Maternal smoking during pregnancy - No | 1 (Reference) | NA | 1.49 (1.1, 2) | **0.009** | 1.49 (1.1, 2) | **0.009** |
| Maternal smoking during pregnancy - Yes | 1.54 (0.81, 2.91) | 0.184 | 1.34 (0.65, 2.74) | 0.436 | 0.87 (0.36, 2.07) | 0.762 |
| Effect of maternal smoking during pregnancy within the strata of maternal pre-pregnancy weight status | 1.54 (0.81, 2.91) | 0.184 | 0.9 (0.45, 1.82) | 0.781 |  |  |
| Multiplicative scale | 0.58 (0.23, 1.46) | 0.249 |  |  |  |  |
| Additive scale (RERI) | -0.69 (-2.26, 0.77) | NA |  |  |  |  |
|  |  |  |  |  |  |  |
| **Year-3 AROW** |  |  |  |  |  |  |
| Maternal smoking during pregnancy - No | 1 (Reference) | NA | 1.71 (1.3, 2.25) | **<0.001** | 1.71 (1.3, 2.25) | **<0.001** |
| Maternal smoking during pregnancy - Yes | 2.09 (1.15, 3.8) | **0.016** | 2.55 (1.26, 5.16) | **0.009** | 1.22 (0.52, 2.86) | 0.657 |
| Effect of maternal smoking during pregnancy within the strata of maternal pre-pregnancy weight status | 2.09 (1.15, 3.8) | **0.016** | 1.49 (0.74, 2.99) | 0.265 |  |  |
| Multiplicative scale | 0.71 (0.29, 1.74) | 0.457 |  |  |  |  |
| Additive scale (RERI) | -0.25 (-2.34, 2.36) | NA |  |  |  |  |

mNW: maternal normal weight; mOWOB: maternal overweight or obese. RERI: relative excess risk due to interaction.

Table S5. Comparisons of the effects of maternal smoking during pregnancy and postnatal home smoke exposure on childhood BMI outcomes.

|  |  | **Prenatal exposure** | |  | **Postnatal exposure** | |  | **Interaction** | |
| --- | --- | --- | --- | --- | --- | --- | --- | --- | --- |
| Outcome | Postnatal exposure | Beta (95% CI) | *P* value |  | Beta (95% CI) | *P* value |  | Beta (95% CI) | *P* value |
| Year-1 BMI z-score | 3 months | 0.34 (0.05, 0.62) | **0.022** |  | 0.13 (-0.05, 0.31) | 0.147 |  | -0.03 (-0.44, 0.37) | 0.872 |
|  | 6 months | 0.33 (0.04, 0.63) | **0.027** |  | 0.13 (-0.11, 0.36) | 0.296 |  | 0 (-0.46, 0.47) | 0.992 |
|  | 1 year | 0.25 (-0.09, 0.58) | 0.149 |  | 0.03 (-0.17, 0.22) | 0.784 |  | 0.15 (-0.31, 0.61) | 0.528 |
| Year-3 BMI z-score | 3 months | 0.36 (0.07, 0.64) | **0.014** |  | 0.16 (-0.01, 0.33) | 0.07 |  | 0.01 (-0.4, 0.41) | 0.974 |
|  | 6 months | 0.39 (0.11, 0.68) | **0.007** |  | 0.18 (-0.04, 0.4) | 0.112 |  | -0.13 (-0.59, 0.32) | 0.56 |
|  | 1 year | 0.31 (-0.03, 0.64) | 0.075 |  | 0.14 (-0.05, 0.33) | 0.142 |  | 0.05 (-0.4, 0.51) | 0.823 |
|  | 3 years | 0.33 (-0.01, 0.67) | 0.057 |  | 0.14 (-0.03, 0.3) | 0.112 |  | -0.1 (-0.57, 0.38) | 0.691 |

Table S6. Associations between concentrations of nicotine metabolites at 3 months and year-1 and year-3 weight outcomes.

|  | Year-1 outcome | | | | | |  | Year-3 outcome | | | | | |
| --- | --- | --- | --- | --- | --- | --- | --- | --- | --- | --- | --- | --- | --- |
|  | BMI z-score | |  | Metabolite concentration (log2) | | |  | BMI z-score | |  | Metabolite concentration (log2) | | |
|  | Pearson's Rho | P value |  | Normal weight | AROW | t test p value |  | Pearson's Rho | P value |  | Normal weight | AROW | t test p value |
| Cotinine | 0.091 | <0.001 |  | -3.01 (2.35) | -2.72 (2.66) | 0.086 |  | 0.097 | <0.001 |  | -3 (2.38) | -2.87 (2.51) | 0.365 |
| *Trans-3'-* hydroxycotinine | 0.082 | 0.002 |  | -2.09 (2.5) | -1.79 (2.89) | 0.103 |  | 0.127 | <0.001 |  | -2.15 (2.46) | -1.84 (2.73) | 0.053 |

Table S7. Gut microbiota alpha diversity indices at 3 and 12 months by exposure to maternal smoking during pregnancy.

|  | 3-month microbiota | | | |  | 12-month microbiota | | | |  | Fixed effect from 3 months to 12 months (Yes vs. No) |  |  |
| --- | --- | --- | --- | --- | --- | --- | --- | --- | --- | --- | --- | --- | --- |
|  | Maternal smoking during pregnancy | |  |  |  | Maternal smoking during pregnancy | |  |  |  |  |  |  |
| Alpha diversity index | No [Median (IRQ)] | Yes [Median (IRQ)] | *P* | FDR |  | No [Median (IRQ)] | Yes [Median (IRQ)] | *P* | FDR |  |  | *P* | FDR |
| **Bacteria (overall)** |  |  |  |  |  |  |  |  |  |  |  |  |  |
| Chao1 | -0.03 (-0.69, 0.65) | 0.35 (-0.28, 0.79) | 0.001 | **0.004** |  | -0.03 (-0.7, 0.64) | 0.29 (-0.4, 1.04) | <0.001 | **0.005** |  | 0.33 (0.176, 0.479) | <0.001 | **<0.001** |
| Observed ASVs | -0.05 (-0.68, 0.66) | 0.31 (-0.28, 0.79) | 0.001 | **0.004** |  | -0.02 (-0.68, 0.64) | 0.27 (-0.44, 0.98) | <0.001 | **0.005** |  | 0.32 (0.172, 0.477) | <0.001 | **<0.001** |
| Shannon | -0.03 (-0.7, 0.66) | 0.3 (-0.24, 0.88) | <0.001 | **0.002** |  | -0.01 (-0.68, 0.66) | 0.13 (-0.54, 0.83) | 0.279 | 0.435 |  | 0.21 (0.057, 0.358) | 0.007 | **0.014** |
| Simpson | -0.03 (-0.69, 0.65) | 0.31 (-0.47, 0.94) | 0.001 | **0.004** |  | 0 (-0.67, 0.66) | 0.03 (-0.7, 0.77) | 0.711 | 0.847 |  | 0.16 (0.008, 0.305) | 0.039 | 0.057 |
| Faith’s phylogenetic diversity | -0.03 (-0.71, 0.63) | 0.44 (-0.23, 1.12) | <0.001 | **<0.001** |  | -0.02 (-0.7, 0.65) | 0.28 (-0.41, 0.97) | 0.001 | **0.005** |  | 0.41 (0.259, 0.559) | <0.001 | **<0.001** |
| **Actinobacteria** |  |  |  |  |  |  |  |  |  |  |  |  |  |
| Chao1 | -0.14 (-0.79, 0.74) | 0.12 (-0.44, 0.64) | 0.018 | **0.032** |  | 0.1 (-0.61, 0.68) | 0.1 (-0.61, 0.68) | 0.045 | 0.08 |  | 0.21 (0.062, 0.355) | 0.005 | **0.011** |
| Observed ASVs | -0.12 (-0.78, 0.58) | 0.13 (-0.43, 0.58) | 0.015 | **0.028** |  | 0.12 (-0.6, 0.74) | 0.12 (-0.6, 0.74) | 0.043 | 0.08 |  | 0.21 (0.062, 0.356) | 0.005 | **0.011** |
| Shannon | 0 (-0.68, 0.66) | -0.02 (-0.55, 0.85) | 0.183 | 0.285 |  | -0.01 (-0.67, 0.66) | 0.19 (-0.69, 0.74) | 0.353 | 0.52 |  | 0.12 (-0.031, 0.271) | 0.118 | 0.156 |
| Simpson | 0 (-0.68, 0.65) | 0.02 (-0.59, 0.81) | 0.373 | 0.443 |  | -0.01 (-0.67, 0.67) | 0.12 (-0.62, 0.68) | 0.552 | 0.691 |  | 0.08 (-0.064, 0.233) | 0.265 | 0.315 |
| Faith’s phylogenetic diversity | -0.03 (-0.7, 0.67) | 0.31 (-0.4, 0.87) | 0.002 | **0.004** |  | -0.02 (-0.69, 0.65) | 0.24 (-0.39, 0.88) | 0.005 | **0.013** |  | 0.26 (0.11, 0.409) | <0.001 | **0.002** |
| **Bacteroidetes** |  |  |  |  |  |  |  |  |  |  |  |  |  |
| Chao1 | -0.01 (-0.72, 0.67) | 0.1 (-0.49, 0.72) | 0.261 | 0.344 |  | -0.06 (-0.69, 0.63) | 0.29 (-0.51, 0.88) | 0.012 | **0.027** |  | 0.18 (0.016, 0.336) | 0.031 | 0.051 |
| Observed ASVs | 0.01 (-0.71, 0.68) | 0.11 (-0.65, 0.71) | 0.28 | 0.35 |  | -0.02 (-0.65, 0.66) | 0.23 (-0.48, 0.86) | 0.017 | **0.035** |  | 0.17 (0.012, 0.332) | 0.035 | 0.055 |
| Shannon | -0.01 (-0.66, 0.67) | 0.15 (-0.71, 0.73) | 0.615 | 0.699 |  | 0 (-0.67, 0.66) | -0.06 (-0.85, 0.84) | 0.821 | 0.933 |  | 0.02 (-0.135, 0.175) | 0.799 | 0.908 |
| Simpson | 0 (-0.67, 0.67) | 0.06 (-0.71, 0.68) | 0.766 | 0.833 |  | 0 (-0.66, 0.67) | 0.05 (-0.84, 0.69) | 0.948 | 0.948 |  | 0 (-0.15, 0.153) | 0.986 | 0.986 |
| Faith’s phylogenetic diversity | -0.01 (-0.68, 0.67) | 0.09 (-0.53, 0.73) | 0.228 | 0.317 |  | -0.02 (-0.71, 0.64) | 0.26 (-0.36, 0.93) | 0.003 | **0.008** |  | 0.22 (0.067, 0.371) | 0.005 | **0.011** |
| **Firmicutes** |  |  |  |  |  |  |  |  |  |  |  |  |  |
| Chao1 | -0.06 (-0.68, 0.65) | 0.4 (-0.14, 0.92) | <0.001 | **<0.001** |  | -0.02 (-0.69, 0.64) | 0.43 (-0.37, 0.97) | 0.001 | **0.005** |  | 0.39 (0.234, 0.539) | <0.001 | **<0.001** |
| Observed ASVs | -0.03 (-0.76, 0.63) | 0.41 (-0.11, 0.89) | <0.001 | **<0.001** |  | -0.04 (-0.7, 0.64) | 0.43 (-0.34, 0.91) | 0.001 | **0.005** |  | 0.39 (0.232, 0.539) | <0.001 | **<0.001** |
| Shannon | -0.03 (-0.69, 0.64) | 0.38 (-0.44, 0.92) | <0.001 | **0.003** |  | -0.03 (-0.7, 0.64) | 0.35 (-0.29, 0.91) | 0.001 | **0.005** |  | 0.3 (0.146, 0.45) | <0.001 | **<0.001** |
| Simpson | -0.03 (-0.69, 0.64) | 0.34 (-0.46, 0.91) | 0.003 | **0.007** |  | -0.02 (-0.69, 0.65) | 0.26 (-0.38, 0.9) | 0.011 | **0.027** |  | 0.24 (0.096, 0.393) | 0.001 | **0.003** |
| Faith’s phylogenetic diversity | -0.05 (-0.72, 0.62) | 0.54 (-0.06, 1.19) | <0.001 | **<0.001** |  | -0.02 (-0.69, 0.65) | 0.34 (-0.31, 1.05) | 0.001 | **0.005** |  | 0.47 (0.319, 0.628) | <0.001 | **<0.001** |
| **Proteobacteria** |  |  |  |  |  |  |  |  |  |  |  |  |  |
| Chao1 | 0.03 (-0.58, 0.7) | 0.03 (-0.58, 0.56) | 0.932 | 0.932 |  | -0.13 (-0.87, 0.78) | -0.13 (-0.48, 0.49) | 0.911 | 0.948 |  | 0 (-0.149, 0.139) | 0.946 | 0.986 |
| Observed ASVs | 0.05 (-0.57, 0.73) | 0.05 (-0.57, 0.58) | 0.913 | 0.932 |  | -0.11 (-0.86, 0.53) | -0.11 (-0.47, 0.53) | 0.946 | 0.948 |  | 0 (-0.149, 0.139) | 0.949 | 0.986 |
| Shannon | -0.02 (-0.7, 0.65) | 0.2 (-0.4, 0.88) | 0.023 | **0.038** |  | 0 (-0.68, 0.67) | -0.03 (-0.59, 0.88) | 0.394 | 0.535 |  | 0.13 (-0.015, 0.279) | 0.078 | 0.109 |
| Simpson | -0.02 (-0.7, 0.64) | 0.26 (-0.52, 0.91) | 0.005 | **0.011** |  | 0 (-0.68, 0.66) | -0.01 (-0.61, 1) | 0.406 | 0.535 |  | 0.16 (0.018, 0.31) | 0.027 | **0.049** |
| Faith’s phylogenetic diversity | -0.01 (-0.68, 0.66) | 0.08 (-0.54, 0.75) | 0.212 | 0.312 |  | -0.01 (-0.68, 0.66) | 0.07 (-0.48, 0.72) | 0.235 | 0.391 |  | 0.09 (-0.049, 0.234) | 0.2 | 0.25 |

Table S8. Correlations between microbial abundance and detailed maternal smoking categories during pregnancy.

| Age | Differentially abundant taxon | Direction of enrichment | Spearman's Rho | *P* value | FDR |
| --- | --- | --- | --- | --- | --- |
| 3 months | k__Bacteria.p__Firmicutes | Exposed | 0.08 | 0.001 | 0.004 |
| 3 months | k__Bacteria.p__Firmicutes.c__Clostridia | Exposed | 0.09 | 0.001 | 0.002 |
| 3 months | k__Bacteria.p__Firmicutes.c__Clostridia.o__Lachnospirales | Exposed | 0.11 | <0.001 | <0.001 |
| 3 months | k__Bacteria.p__Firmicutes.c__Clostridia.o__Lachnospirales.f__Lachnospiraceae | Exposed | 0.11 | <0.001 | <0.001 |
| 3 months | k__Bacteria.p__Firmicutes.c__Clostridia.o__Lachnospirales.f__Lachnospiraceae.g__Lachnoclostridium | Exposed | 0.09 | <0.001 | 0.001 |
| 3 months | k__Bacteria.p__Firmicutes.c__Clostridia.o__Lachnospirales.f__Lachnospiraceae.g__.Ruminococcus._gnavus_group | Exposed | 0.11 | <0.001 | <0.001 |
| 3 months | k__Bacteria.p__Firmicutes.c__Clostridia.o__Lachnospirales.f__Lachnospiraceae.g__.Ruminococcus._torques_group | Exposed | 0.07 | 0.006 | 0.017 |
| 3 months | k__Bacteria.p__Firmicutes.c__Clostridia.o__Oscillospirales | Exposed | 0.16 | <0.001 | <0.001 |
| 3 months | k__Bacteria.p__Firmicutes.c__Clostridia.o__Oscillospirales.f__Oscillospiraceae | Exposed | 0.16 | <0.001 | <0.001 |
| 3 months | k__Bacteria.p__Firmicutes.c__Clostridia.o__Oscillospirales.f__Oscillospiraceae.g__Flavonifractor | Exposed | 0.15 | <0.001 | <0.001 |
| 3 months | k__Bacteria.p__Firmicutes.c__Clostridia.o__Oscillospirales.f__Ruminococcaceae.g__Incertae_Sedis | Exposed | 0.16 | <0.001 | <0.001 |
| 3 months | k__Bacteria.p__Firmicutes.c__Clostridia.o__Oscillospirales.f__Ruminococcaceae.g__UBA1819 | Exposed | 0.06 | 0.018 | 0.042 |
| 3 months | k__Bacteria.p__Firmicutes.c__Clostridia.o__Peptostreptococcales.Tissierellales | Exposed | 0.14 | <0.001 | <0.001 |
| 3 months | k__Bacteria.p__Firmicutes.c__Clostridia.o__Peptostreptococcales.Tissierellales.f__Peptostreptococcaceae | Exposed | 0.13 | <0.001 | <0.001 |
| 3 months | k__Bacteria.p__Firmicutes.c__Clostridia.o__Peptostreptococcales.Tissierellales.f__Peptostreptococcaceae.g__Clostridioides | Exposed | 0.09 | <0.001 | 0.002 |
| 3 months | k__Bacteria.p__Firmicutes.c__Negativicutes | Exposed | 0.09 | <0.001 | 0.002 |
| 3 months | k__Bacteria.p__Firmicutes.c__Negativicutes.o__Veillonellales.Selenomonadales | Exposed | 0.08 | 0.003 | 0.008 |
| 3 months | k__Bacteria.p__Firmicutes.c__Negativicutes.o__Veillonellales.Selenomonadales.f__Veillonellaceae | Exposed | 0.07 | 0.003 | 0.010 |
| 3 months | k__Bacteria.p__Firmicutes.c__Negativicutes.o__Veillonellales.Selenomonadales.f__Veillonellaceae.g__Megasphaera | Exposed | 0.15 | <0.001 | <0.001 |
| 3 months | k__Bacteria.p__Proteobacteria.c__Gammaproteobacteria.o__Burkholderiales | Exposed | 0.07 | 0.004 | 0.012 |
| 3 months | k__Bacteria.p__Proteobacteria.c__Gammaproteobacteria.o__Burkholderiales.f__Sutterellaceae | Exposed | 0.09 | <0.001 | 0.001 |
| 3 months | k__Bacteria.p__Proteobacteria.c__Gammaproteobacteria.o__Burkholderiales.f__Sutterellaceae.g__Sutterella | Exposed | 0.10 | <0.001 | <0.001 |
| 3 months | k__Bacteria.p__Actinobacteriota.c__Actinobacteria.o__Micrococcales | Not exposed | -0.05 | 0.052 | 0.105 |
| 3 months | k__Bacteria.p__Actinobacteriota.c__Actinobacteria.o__Micrococcales.f__Micrococcaceae | Not exposed | -0.05 | 0.043 | 0.090 |
| 3 months | k__Bacteria.p__Actinobacteriota.c__Actinobacteria.o__Micrococcales.f__Micrococcaceae.g__Rothia | Not exposed | -0.05 | 0.045 | 0.092 |
| 3 months | k__Bacteria.p__Firmicutes.c__Clostridia.o__Lachnospirales.f__Lachnospiraceae.g__Blautia | Not exposed | 0.07 | 0.007 | 0.018 |
| 3 months | k__Bacteria.p__Firmicutes.c__Clostridia.o__Oscillospirales.f__Ruminococcaceae | Not exposed | 0.13 | <0.001 | <0.001 |
| 3 months | k__Bacteria.p__Proteobacteria.c__Gammaproteobacteria.o__Pasteurellales | Not exposed | -0.10 | <0.001 | 0.001 |
| 3 months | k__Bacteria.p__Proteobacteria.c__Gammaproteobacteria.o__Pasteurellales.f__Pasteurellaceae | Not exposed | -0.10 | <0.001 | 0.001 |
| 3 months | k__Bacteria.p__Proteobacteria.c__Gammaproteobacteria.o__Pasteurellales.f__Pasteurellaceae.g__Haemophilus | Not exposed | -0.10 | <0.001 | 0.001 |
| 12 months | k__Bacteria.p__Desulfobacterota | Exposed | 0.08 | 0.002 | 0.016 |
| 12 months | k__Bacteria.p__Desulfobacterota.c__Desulfovibrionia | Exposed | 0.08 | 0.003 | 0.018 |
| 12 months | k__Bacteria.p__Desulfobacterota.c__Desulfovibrionia.o__Desulfovibrionales | Exposed | 0.08 | 0.003 | 0.018 |
| 12 months | k__Bacteria.p__Desulfobacterota.c__Desulfovibrionia.o__Desulfovibrionales.f__Desulfovibrionaceae | Exposed | 0.08 | 0.003 | 0.018 |
| 12 months | k__Bacteria.p__Desulfobacterota.c__Desulfovibrionia.o__Desulfovibrionales.f__Desulfovibrionaceae.g__Bilophila | Exposed | 0.08 | 0.002 | 0.017 |
| 12 months | k__Bacteria.p__Firmicutes.c__Bacilli.o__Erysipelotrichales.f__Erysipelatoclostridiaceae | Exposed | 0.03 | 0.274 | 0.488 |
| 12 months | k__Bacteria.p__Firmicutes.c__Clostridia.o__Oscillospirales.f__Butyricicoccaceae | Exposed | 0.05 | 0.074 | 0.219 |
| 12 months | k__Bacteria.p__Firmicutes.c__Clostridia.o__Oscillospirales.f__Butyricicoccaceae.g__Butyricicoccus | Exposed | 0.04 | 0.108 | 0.274 |
| 12 months | k__Bacteria.p__Firmicutes.c__Clostridia.o__Oscillospirales.f__Oscillospiraceae | Exposed | 0.07 | 0.010 | 0.051 |
| 12 months | k__Bacteria.p__Firmicutes.c__Clostridia.o__Oscillospirales.f__Oscillospiraceae.g__Colidextribacter | Exposed | 0.15 | <0.001 | <0.001 |
| 12 months | k__Bacteria.p__Firmicutes.c__Clostridia.o__Oscillospirales.f__Oscillospiraceae.g__Flavonifractor | Exposed | 0.07 | 0.013 | 0.061 |
| 12 months | k__Bacteria.p__Firmicutes.c__Clostridia.o__Oscillospirales.f__Ruminococcaceae.g__Incertae_Sedis | Exposed | 0.09 | <0.001 | 0.005 |
| 12 months | k__Bacteria.p__Firmicutes.c__Clostridia.o__Oscillospirales.f__Ruminococcaceae.g__UBA1819 | Exposed | 0.15 | <0.001 | <0.001 |
| 12 months | k__Bacteria.p__Proteobacteria.c__Gammaproteobacteria.o__Burkholderiales.f__Sutterellaceae.g__Sutterella | Exposed | 0.09 | 0.001 | 0.012 |
| 12 months | k__Bacteria.p__Firmicutes.c__Clostridia.o__Lachnospirales.f__Lachnospiraceae.g__Lachnospira | Not exposed | -0.04 | 0.103 | 0.274 |
| 12 months | k__Bacteria.p__Firmicutes.c__Negativicutes.o__Veillonellales.Selenomonadales | Not exposed | -0.04 | 0.111 | 0.274 |
| 12 months | k__Bacteria.p__Firmicutes.c__Negativicutes.o__Veillonellales.Selenomonadales.f__Veillonellaceae | Not exposed | -0.06 | 0.036 | 0.134 |
| 12 months | k__Bacteria.p__Firmicutes.c__Negativicutes.o__Veillonellales.Selenomonadales.f__Veillonellaceae.g__Veillonella | Not exposed | -0.08 | 0.002 | 0.016 |
| 12 months | k__Bacteria.p__Proteobacteria.c__Gammaproteobacteria.o__Pasteurellales | Not exposed | -0.07 | 0.008 | 0.041 |
| 12 months | k__Bacteria.p__Proteobacteria.c__Gammaproteobacteria.o__Pasteurellales.f__Pasteurellaceae | Not exposed | -0.07 | 0.008 | 0.041 |
| 12 months | k__Bacteria.p__Proteobacteria.c__Gammaproteobacteria.o__Pasteurellales.f__Pasteurellaceae.g__Haemophilus | Not exposed | -0.07 | 0.009 | 0.046 |

Table S9. Joint mediation effects of selected mediators.

|  | Year-1 BMI z-score | Year-1 AROW | Year-3 BMI z-score | Year-3 AROW |
| --- | --- | --- | --- | --- |
| 3M microbiota | 153.2% (-95.3%, 588.0%) | 57.9% (-165.5%, 595.5%) | **24.2% (8.3%, 54.5%)** | **18.6% (3.2%, 41.7%)** |
| 12M microbiota | N.A. | N.A. | **13.9% (0.6%, 34.6%)** | 15.8% (-1.5%, 60.5%) |

Note: In this analysis, Firmicutes Faith’s phylogenetic diversity index, Oscillospiraceae, Lachnospiraceae and *Haemophilus* were included. Firmicutes Chao1 index was not included because it was higly correlated with the Firmicutes Faith’s phylogenetic diversity index; while *Flavonifractor* was not included as its family Oscillospiraceae has a larger effect size in the separate model. Maternal race/ethnicity, socioeconomic status, maternal pre-pregnancy BMI, prenatal and postnatal pet exposure, breastfeeding status at three months, solid food introduction at three months, household disinfectant use, prenatal diet calories, infant sex, birth mode, antibiotic use and the age of sample collection were adjusted in the model.

Table S10. Associations between gut metabolites at age 3 months and BMI z-scores at ages 1 and 3 years.

|  | **Maternal pre-pregnancy BMI** | | |  | **Year-1 BMI z-scores** | | |  | **Year-3 BMI z-scores** | | |
| --- | --- | --- | --- | --- | --- | --- | --- | --- | --- | --- | --- |
| **Metabolite** | **Spearman's Rho** | ***P* value** | **FDR** |  | **Spearman's Rho** | ***P* value** | **FDR** |  | **Spearman's Rho** | ***P* value** | **FDR** |
| Pyruvate | -0.16 | <0.001 | **0.001** |  | -0.07 | 0.074 | 0.304 |  | -0.16 | <0.001 | **0.005** |
| Propyleneglycol | -0.19 | <0.001 | **<0.001** |  | -0.10 | 0.025 | 0.183 |  | -0.16 | <0.001 | **0.005** |
| Isovalerate | 0.2 | <0.001 | **<0.001** |  | 0.08 | 0.037 | 0.204 |  | 0.10 | 0.014 | 0.085 |
| Butyrate | 0.18 | <0.001 | **<0.001** |  | 0.13 | 0.002 | **0.055** |  | 0.14 | 0.001 | **0.011** |
| Isobutyrate | 0.19 | <0.001 | **<0.001** |  | 0.10 | 0.014 | 0.183 |  | 0.10 | 0.025 | 0.116 |
| Lactate | -0.12 | 0.004 | **0.017** |  | -0.04 | 0.384 | 0.662 |  | -0.11 | 0.015 | 0.085 |
| Glucose | -0.1 | 0.022 | 0.065 |  | -0.02 | 0.674 | 0.820 |  | -0.11 | 0.013 | 0.085 |
| Choline | -0.13 | 0.003 | **0.014** |  | -0.02 | 0.661 | 0.820 |  | -0.09 | 0.055 | 0.203 |
| Propionate | 0.18 | <0.001 | **<0.001** |  | 0.09 | 0.028 | 0.183 |  | 0.07 | 0.119 | 0.326 |
| Creatinine | -0.04 | 0.376 | 0.47 |  | 0.01 | 0.781 | 0.832 |  | -0.07 | 0.116 | 0.326 |
| Tryptophan | 0.11 | 0.005 | **0.019** |  | -0.01 | 0.782 | 0.832 |  | -0.02 | 0.567 | 0.882 |
| Threonine | 0.04 | 0.384 | 0.47 |  | 0.03 | 0.528 | 0.758 |  | 0.03 | 0.570 | 0.882 |
| Glycine | 0.03 | 0.52 | 0.613 |  | 0.02 | 0.696 | 0.820 |  | -0.03 | 0.504 | 0.882 |
| Formate | -0.09 | 0.029 | 0.08 |  | -0.08 | 0.053 | 0.250 |  | -0.10 | 0.031 | 0.127 |
| Succinate | -0.07 | 0.075 | 0.137 |  | 0.00 | 0.973 | 0.973 |  | -0.07 | 0.111 | 0.326 |
| Alanine | 0 | 0.994 | 0.994 |  | 0.05 | 0.242 | 0.569 |  | -0.03 | 0.465 | 0.882 |
| Ethanol | -0.05 | 0.209 | 0.329 |  | 0.03 | 0.442 | 0.663 |  | -0.01 | 0.778 | 0.903 |
| Methanol | -0.05 | 0.24 | 0.344 |  | -0.01 | 0.764 | 0.832 |  | -0.03 | 0.529 | 0.882 |
| Methionine | 0.09 | 0.044 | 0.104 |  | 0.05 | 0.234 | 0.569 |  | 0.02 | 0.689 | 0.903 |
| Uracil | 0.02 | 0.564 | 0.642 |  | 0.03 | 0.405 | 0.662 |  | 0.00 | 0.929 | 0.929 |
| Trimethylamine | 0.07 | 0.093 | 0.162 |  | -0.01 | 0.859 | 0.886 |  | -0.01 | 0.869 | 0.903 |
| Malonate | 0.1 | 0.017 | 0.056 |  | 0.07 | 0.110 | 0.405 |  | 0.04 | 0.397 | 0.882 |
| Glutamate | 0.09 | 0.038 | 0.096 |  | 0.02 | 0.566 | 0.778 |  | -0.02 | 0.705 | 0.903 |
| Aspartate | 0.08 | 0.047 | 0.104 |  | 0.02 | 0.655 | 0.820 |  | 0.02 | 0.578 | 0.882 |
| Valerate | 0.02 | 0.642 | 0.706 |  | 0.03 | 0.421 | 0.662 |  | 0.05 | 0.212 | 0.539 |
| Creatine | -0.01 | 0.823 | 0.873 |  | 0.04 | 0.306 | 0.630 |  | -0.02 | 0.588 | 0.882 |
| Acetate | 0.07 | 0.069 | 0.136 |  | 0.09 | 0.026 | 0.183 |  | 0.01 | 0.754 | 0.903 |
| Fumarate | -0.01 | 0.847 | 0.873 |  | 0.04 | 0.385 | 0.662 |  | 0.01 | 0.865 | 0.903 |
| Isoleucine | 0.07 | 0.113 | 0.186 |  | 0.04 | 0.357 | 0.662 |  | -0.01 | 0.876 | 0.903 |
| Leucine | 0.05 | 0.22 | 0.33 |  | 0.05 | 0.229 | 0.569 |  | -0.01 | 0.844 | 0.903 |
| Phenylalanine | 0.05 | 0.269 | 0.355 |  | 0.05 | 0.227 | 0.569 |  | -0.02 | 0.661 | 0.903 |
| Tyrosine | 0.08 | 0.07 | 0.136 |  | 0.05 | 0.231 | 0.569 |  | 0.03 | 0.524 | 0.882 |
| Valine | 0.05 | 0.268 | 0.355 |  | 0.05 | 0.270 | 0.593 |  | -0.01 | 0.863 | 0.903 |

# Supplementary Figures

**
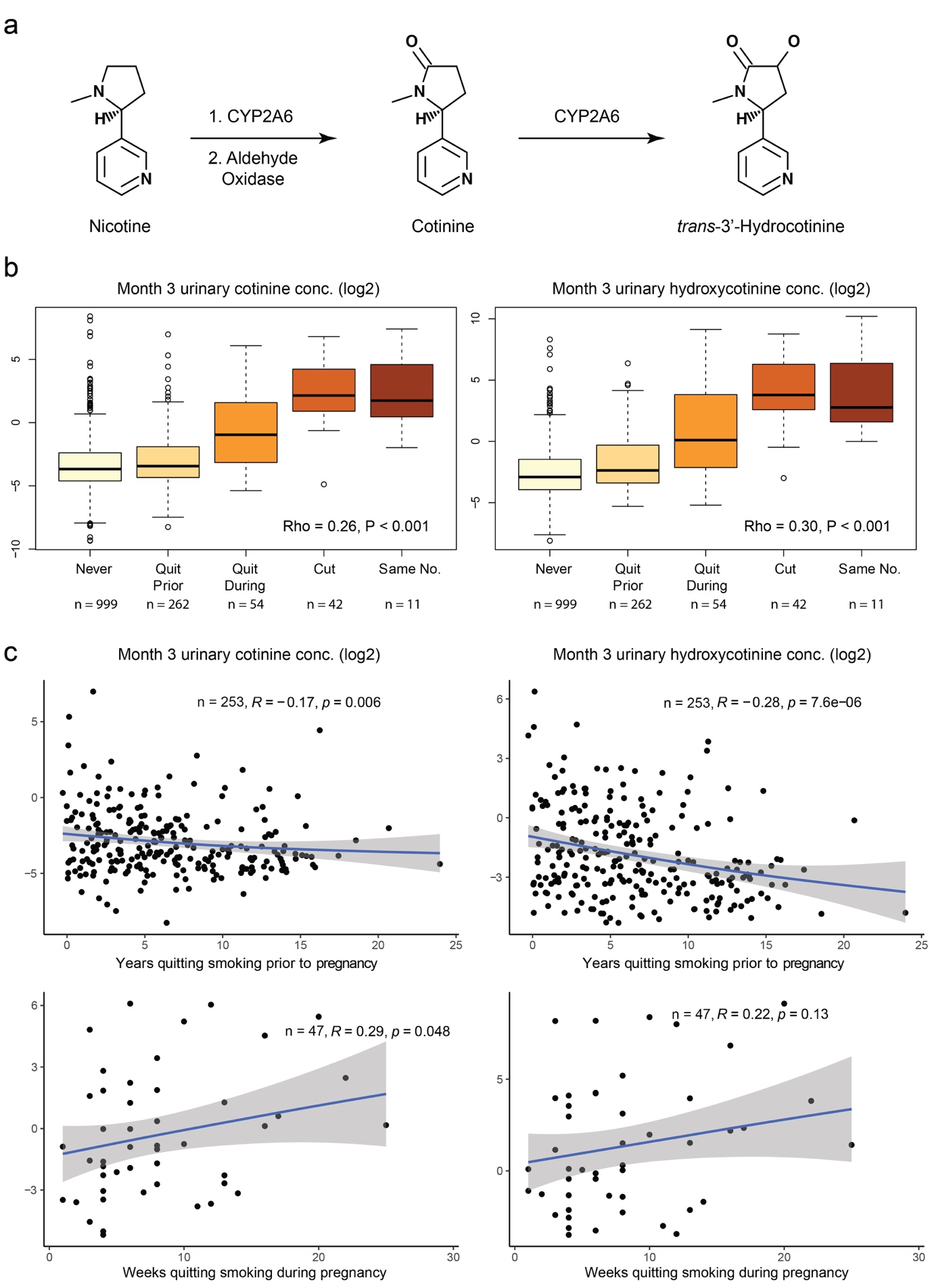
**

Figure S1. Dose-response association between maternal smoking during pregnancy and objective measurements of nicotine metabolites in early infancy.

**(a)** Partial pathways of nicotine metabolism in human body. **(b)** Concentrations of cotinine and trans-3’-hydroxycotinine by maternal smoking during pregnancy. The maternal smoking status during pregnancy was treated as an ordinal variable: never smoker = 1; quit smoking before pregnancy = 2; quit smoking during pregnancy = 3; cut the number of cigarettes during pregnancy = 4; had the same number of cigarettes during pregnancy = 5. **(c)** Correlations between urinary concentrations of nicotine metabolites and years of quitting smoking prior to pregnancy. **(d)** Correlations between urinary concentrations of nicotine metabolites and weeks of pregnancy when quitting smoking. Concentrations were log2 transformed. *Rho* and ***P*** values were given by Spearman’s correlation tests. The regression lines (on the log2-transformed concentrations) were created using the *geom_smooth* function implemented in ggplot2 with the "Generalized Additive Model" (GAM).

Figure S2. Concentrations of urinary nicotine metabolites measured in early infancy by maternal smoking during pregnancy and postnatal home smoke exposure.

*P* values were given by Dunn’s test. *P* values: “***”, <0.001; “**”, <0.01; “*”, < 0.05; “.” < 0.1; “n.s.”, not significant.
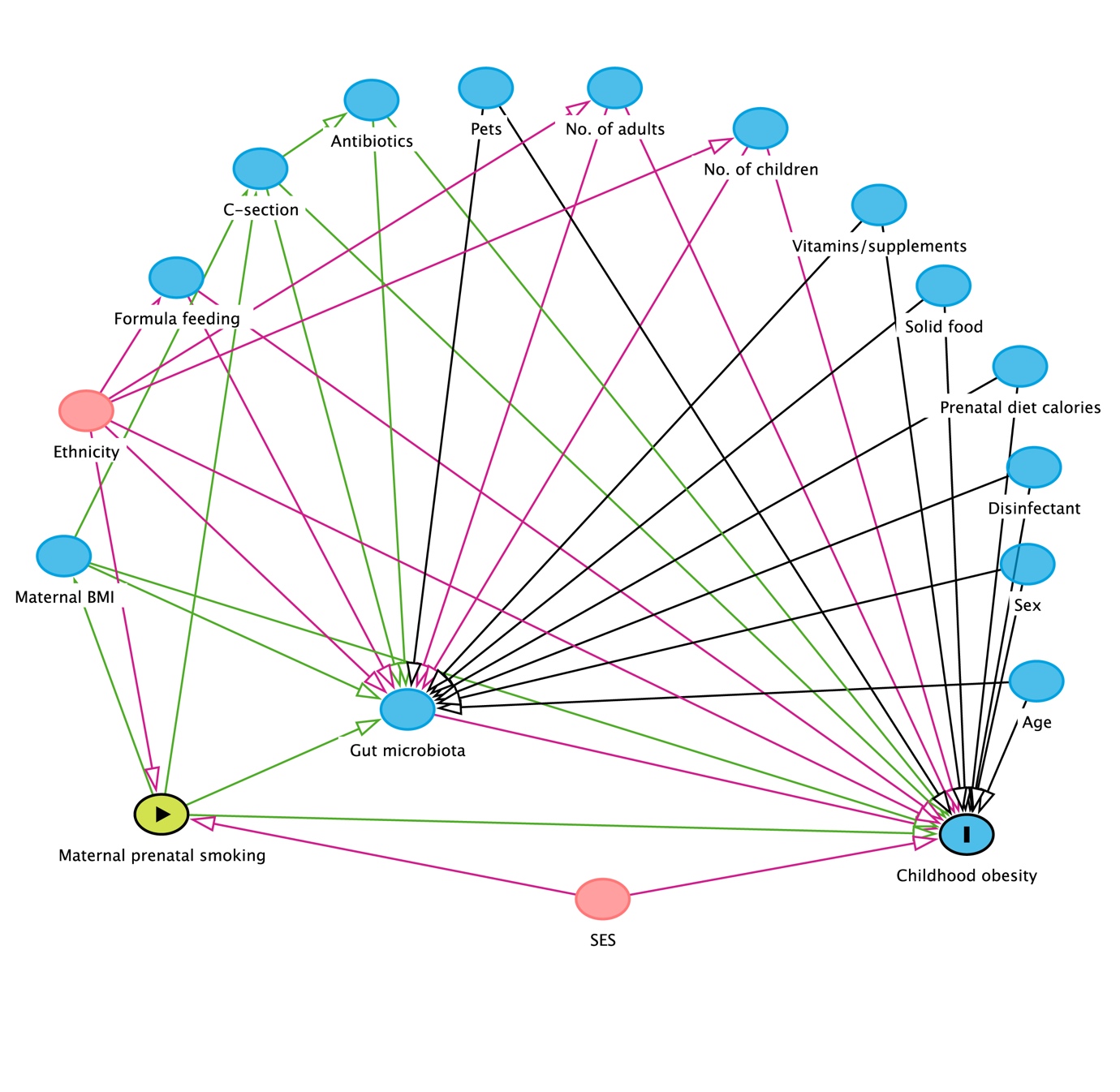
Figure S3. Directed acyclic graph of maternal smoking during pregnancy, gut microbiota, weight outcomes, and potential confounders.

SES, socio-economic status.


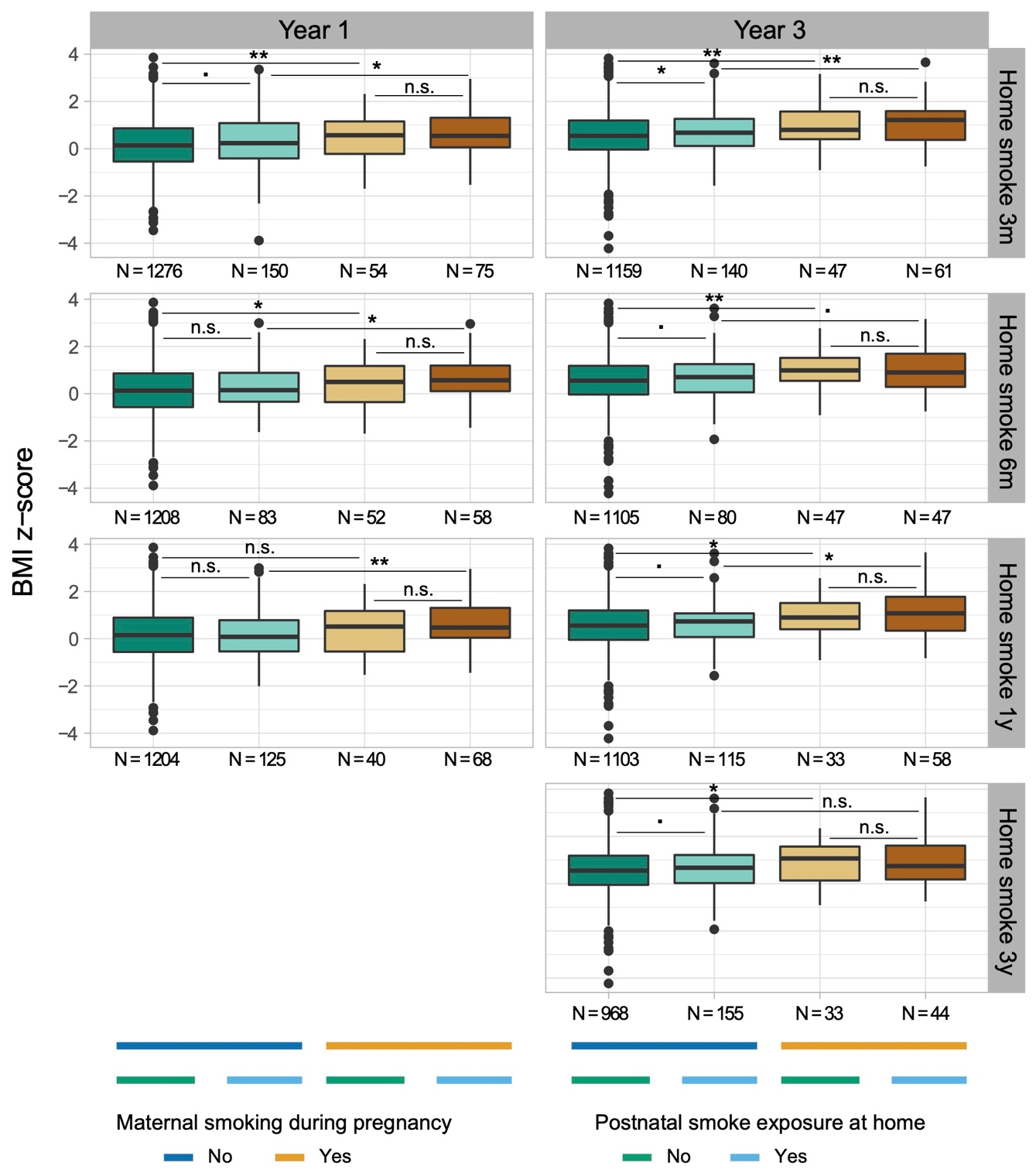


Figure S4. BMI z-scores by maternal smoking during pregnancy and postnatal home smoke exposure.

*P* values were given by Dunn’s test. *P* values: “***”, <0.001; “**”, <0.01; “*”, < 0.05; “.” < 0.1; “n.s.”, not significant.


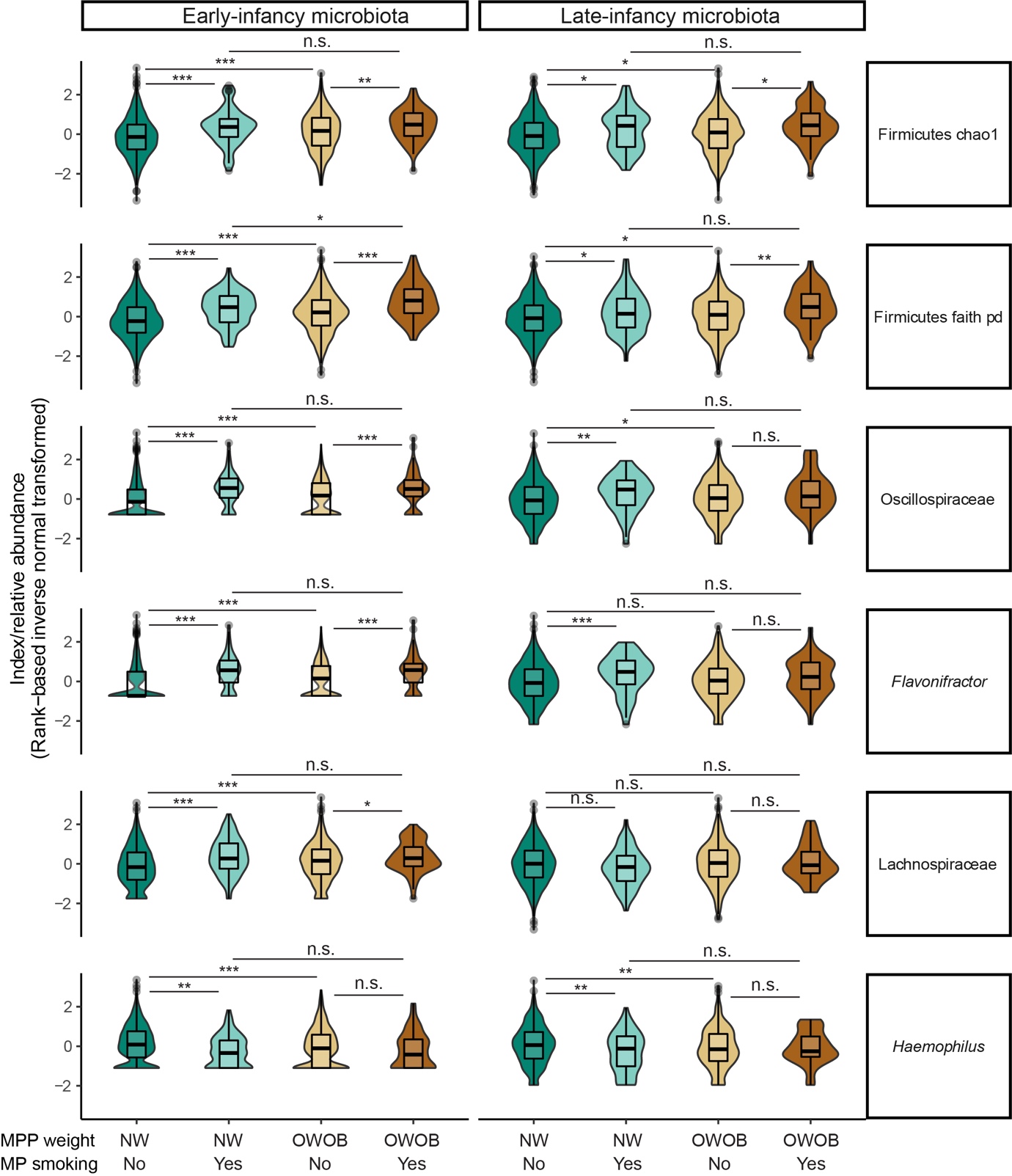


Figure S5. Relative abundance or value of indices of the mediators by maternal smoking during pregnancy and maternal pre-pregnancy weight status.


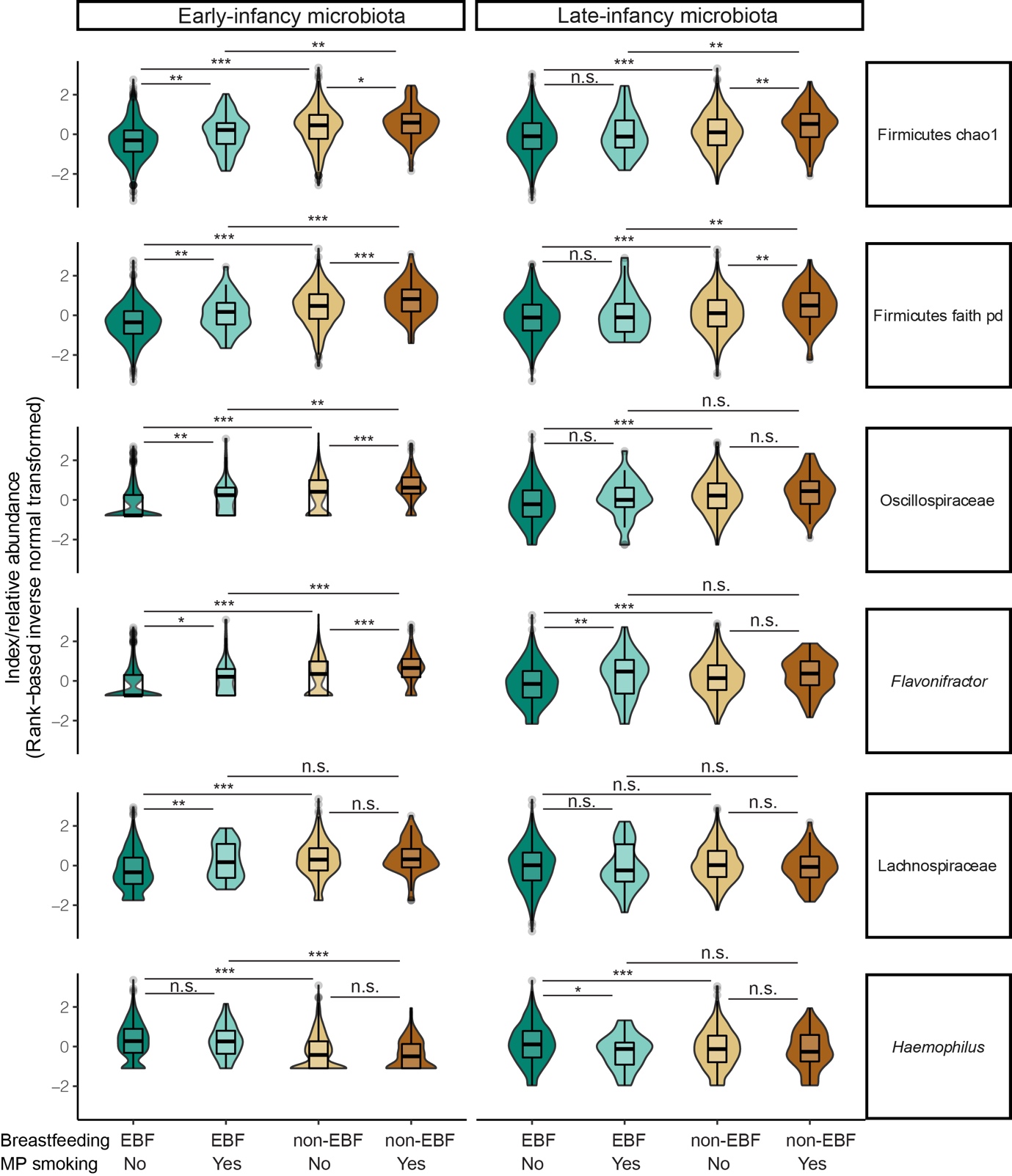


Figure S6. Relative abundance or value of indices of the mediators by maternal smoking during pregnancy and breastfeeding status at 3 months.


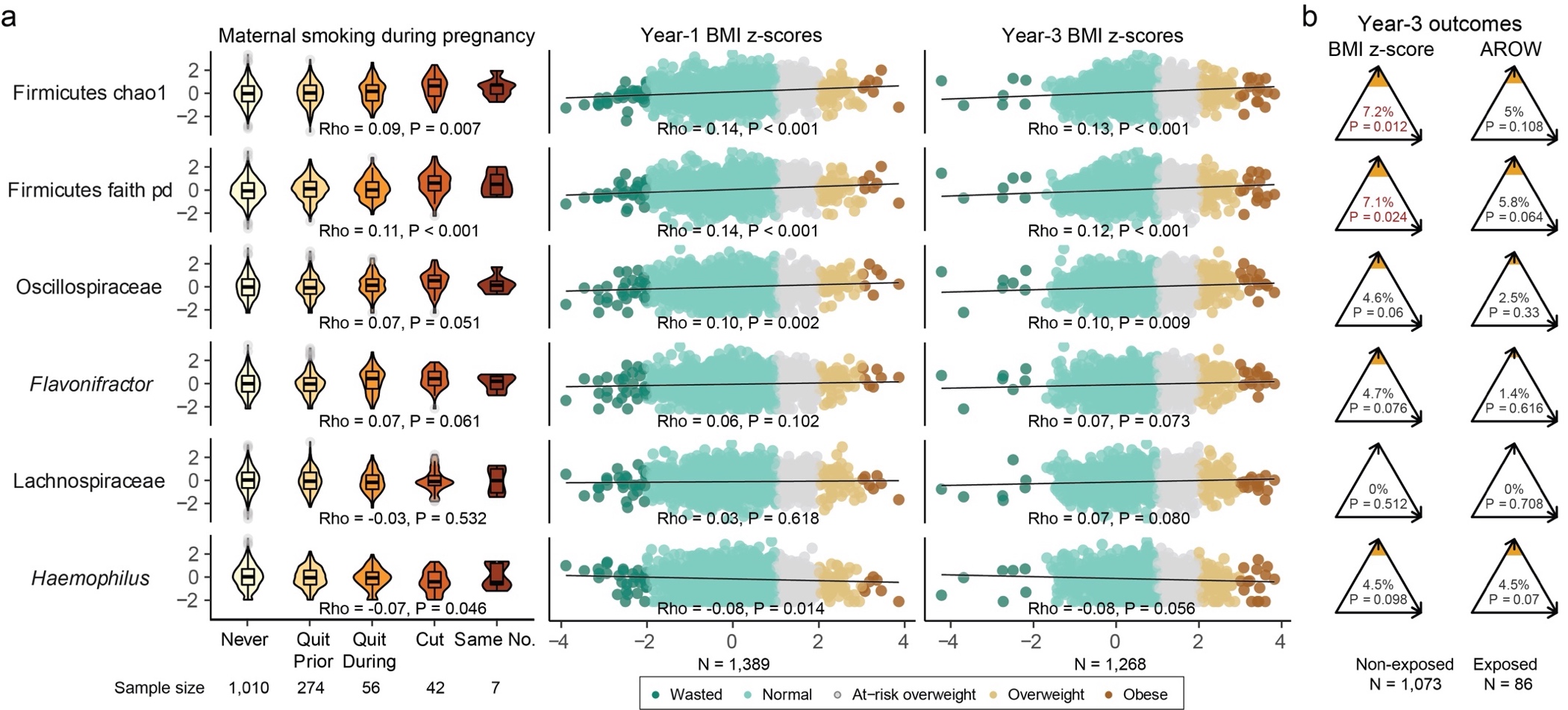


Figure S7. Late-infancy microbial mediators in the pathway from maternal smoking during pregnancy to weight outcomes at 1 and 3 years.

**(a)** Relative abundance or value of indices of the mediators by exposure of maternal smoking during pregnancy, year-1 weight status, and year-3 weight status. The category “wasted” was included here to show the trend, but not included in the downstream analysis because of its small sample size and being out of the scope of the study. *P* values: “***”, <0.001; “**”, <0.01; “*”, < 0.05; “.” < 0.1. *P* values in were FDR-corrected. **(b)** Proportions mediated by the microbial mediators. Maternal pre-pregnancy BMI, maternal ethnicity, formula feeding, and pet exposure were adjusted in the mediator (gut microbiota)-outcome associations, while maternal race/ethnicity and maternal education level was considered in the exposure (maternal smoking during pregnancy)-outcome association.

**
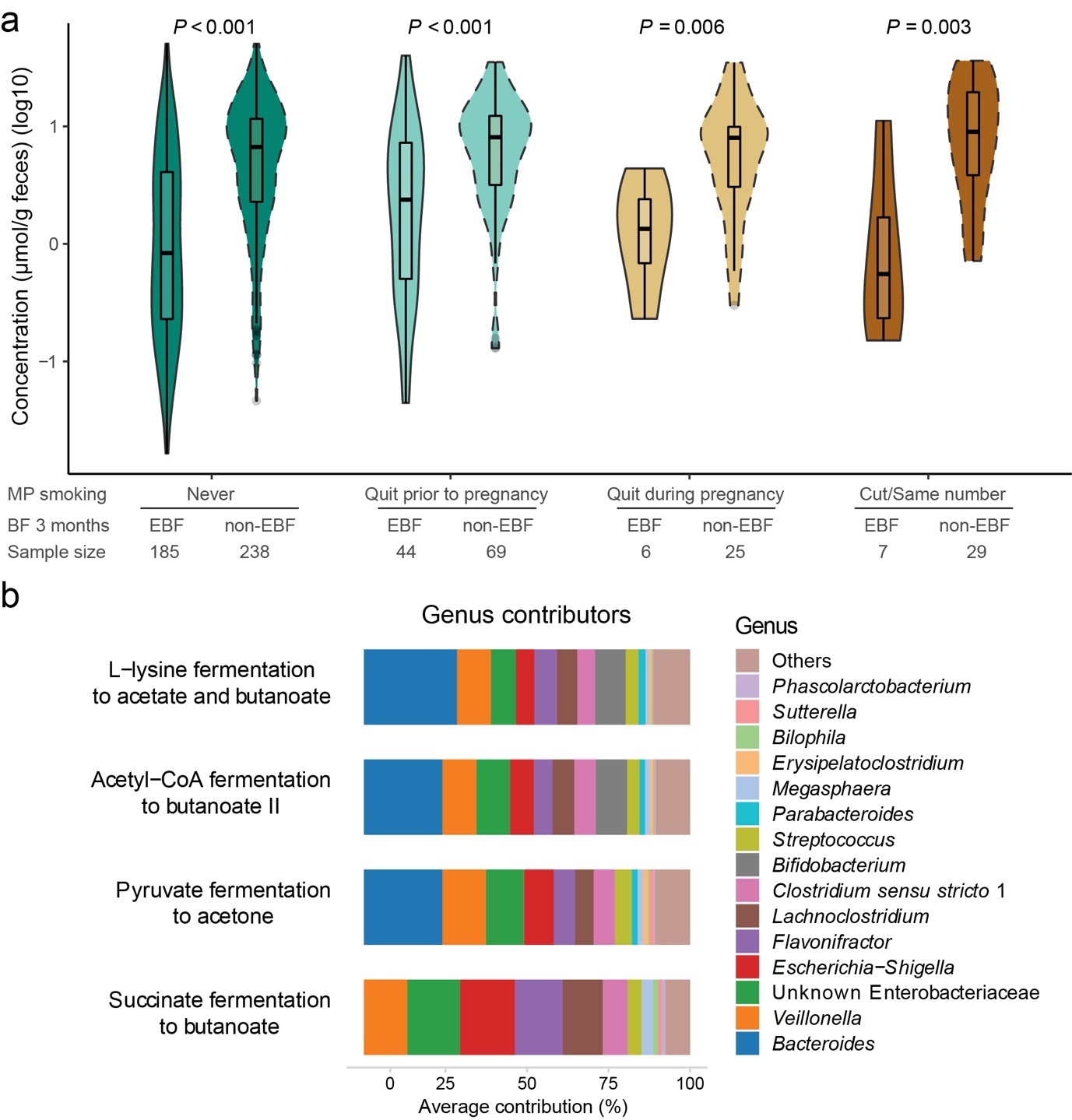
**

Figure S8. Fecal butyrate concentration by breastfeeding status and maternal smoking status during pregnancy and microbial contributors to butyrate-related pathways.

**(a)** Fecal concentration of butyrate in early infancy by breastfeeding status in 3 months and maternal smoking during pregnancy. *P* values were given by Wilcoxon’s rank-sum tests. **(b)** Genus contributors to butyrate, pyruvate and succinate-related microbial pathways. Stacked bars show the average contributions across the cohort.

# Supplementary References

1. Bolyen E, Rideout JR, Dillon MR, Bokulich N, Abnet CC, Al-Ghalith GA, et al. Reproducible, interactive, scalable and extensible microbiome data science using QIIME 2. Nature Biotechnology 2019; 37:852-7.

2. Douglas GM, Maffei VJ, Zaneveld JR, Yurgel SN, Brown JR, Taylor CM, et al. PICRUSt2 for prediction of metagenome functions. Nat Biotechnol 2020; 38:685-8.

3. Caspi R, Billington R, Ferrer L, Foerster H, Fulcher CA, Keseler IM, et al. The MetaCyc database of metabolic pathways and enzymes and the BioCyc collection of pathway/genome databases. Nucleic Acids Res 2016; 44:D471-80.
